# Glycol porphyrin derivatives and temoporfin elicit resistance to photodynamic therapy by different mechanisms

**DOI:** 10.1038/srep44497

**Published:** 2017-03-15

**Authors:** Jarmila Kralova, Michal Kolar, Michal Kahle, Jaroslav Truksa, Sandra Lettlova, Kamila Balusikova, Petr Bartunek

**Affiliations:** 1 CZ-OPENSCREEN: National Infrastructure for Chemical Biology, Institute of Molecular Genetics of the ASCR, v. v. i., Prague, Czech Republic; 2 Laboratory of Genomics and Bioinformatics, Institute of Molecular Genetics of the ASCR, v. v. i., Prague, Czech Republic; 3 Laboratory of Tumor Resistance, Institute of Biotechnology of the ASCR, v. v. i., BIOCEV, Vestec, Prague-West, Czech Republic; 4 Division of Cell and Molecular Biology & Center for Research of Diabetes, Metabolism and Nutrition, Third Faculty of Medicine, Charles University in Prague, Czech Republic

## Abstract

The development of drug resistance is a major problem which often occurs during anticancer chemotherapies. Photodynamic therapy (PDT) has been studied as an alternative treatment modality for drug-resistant tumors, however the question of resistance to PDT and potential cross-resistance with chemotherapy has yet to be fully answered. To investigate the mechanism of resistance to PDT, we developed an *in vitro* experimental model system in a mouse mammary carcinoma cell line 4T1. We used two ethylene glycol derivatives of tetraphenylporphyrin, and tetraphenylchlorin derivative, temoporfin, as photosensitizers (PS). PDT-resistant clones were obtained by exposure to a set concentration of PS followed by irradiation with increasing light doses. PDT resistance to soluble glycol porphyrins was mediated mainly by increased drug efflux through ABCB1 (P-glycoprotein) as we demonstrated by specific ABCB1 knockdown experiments, which in turn rescued the sensitivity of resistant cells to PDT. In contrast, resistance raised to temoporfin, which is generally more lipophilic than glycol porphyrins, elicited mechanism based on sequestration of the drug to lysosomes. The resistance that is acquired from a particular PS could be overcome by using a different PS, which is not susceptible to the same mechanism(s) of resistance. Elucidation of the underlying mechanisms in various types of resistance might facilitate improvements in PDT treatment design.

The efficacy of anticancer chemotherapies is dramatically hampered by multidrug resistance (MDR), i.e. the ability of cancer cells to develop cross-resistance to a range of structurally and functionally unrelated anticancer drugs. Various mechanisms which are involved in MDR have been identified including the enhanced activity of drug pumps, modulation of cellular death pathways, and alteration and repair of target molecules, in addition to less commonly known types. Together, they build a complex network of modifications that mediate an individual MDR phenotype^1^. Resistance to chemotherapy is often circumvented by employing other treatment modalities such as surgery, radiation therapy, immunotherapy, or hormonal therapy. Under some conditions, resistance that has been induced by cytostatic treatment might also be overcome by photodynamic therapy (PDT).

PDT is based on the unique features of a light-absorbing agent (photosensitizer), which selectively accumulates in the tumor and which is then activated by light to trigger oxidative stress and destruction of a cellular target. However, at least in *in vitro* conditions, repeated PDT treatment can induce resistance^2^,^3^,^4^. The mechanisms of PDT resistance may show common features with MDR, thus raising the possibility of occurrence of cross-resistance to both treatments^5^,^6^,^7^. On the other hand, the mechanisms of PDT and chemotherapy may differ, and therefore in some cases no significant cross-resistance has been reported^2^,^4^. In this context, it should be mentioned that in clinical settings, PDT is not always repetitive. Moreover, PDT of wet age-related macular degeneration^8^ and early stage cancers in the upper aerodigestive tract^9^ although repeated was not shown to lead to resistance. Despite these findings, we believe that knowledge acquired regarding the mechanisms of PDT resistance might be useful in combining PDT with classical chemotherapy in refractory cancers^4^.

Some of the common mechanisms of anticancer drug resistance that limit the prolonged and effective use of drugs include the high expression of ATP binding cassette (ABC) efflux transporters such as ABCB1 (multidrug resistance protein 1 - MDR1/P-glycoprotein), ABCC1 (multidrug resistance-associated protein 1 - MRP1), and breast cancer resistance protein ABCG2 (BCRP). ABCB1 is the most prominent and best characterized member of the superfamily of ABC transporters. It is a 170-kDa membrane glycoprotein with a broad spectrum of structurally unrelated substrates which are mostly hydrophobic amphipathic compounds that often possess aromatic rings and a positively charged moiety. In addition, therapeutic drugs, peptides and lipid-like compounds are also found among its substrates. ABCB1 plays a crucial physiological role in the protection of tissues from toxic xenobiotics and endogenous metabolites, and affects the uptake and distribution of many clinically important drugs^1^,^10^,^11^. An X-ray crystal structure of ABCB1 shows that drugs interact within its transmembrane regions by fitting into a large flexible binding pocket that can accommodate several substrate molecules simultaneously^10^,^12^. However, the involvement of ABCB1 in the resistance to PDT in contrast to ABCG2 was not clearly demonstrated^3^. The ABCG2 transporter was shown to be an effective efflux pump of numerous photosensitizers including 5-aminolevulinic acid (ALA)-induced protoporphyrin IX (PpIX), pheophorbide (PhA), chlorin e6 (Ce6), pyropheophorbide a methyl ester (MPPa), 2-(1-hexyloxethyl)-2-devinyl pyropheophorbide-a (HPPH), benzoporphyrin derivative monoacid ring A (BPD-MA), and hypericin^3^,^7^,^13^. ABCG2 is responsible for reducing the intracellular levels of PS below the threshold needed for cell death in tumors treated with PDT, thus leaving resistant cells that have the capability of repopulating the tumor^14^. Results from some studies suggest that ABCG2 binding may be a common mechanism mediating resistance in chemo- and PDT-therapies^15^. Several approaches have been tested to reduce PS efflux in MDR cells, including the application of ABC transporters inhibitors of and implementation of non-substrate PS or PS conjugates^16^. Recently, a new and attractive strategy has been suggested utilizing P-glycoprotein to sequester redox-active and protonable drug substrates into lysosomes, which resulted in catastrophic lysosomal membrane permeabilization and the destruction of MDR cancer cells^17^.

It is expected that the assessment of the cancer MDR profile can be helpful in individualized PS selection and PDT treatment design in future. The structure of PS, a feature assumed to be related to its particular subcellular localization^2^, is believed to be a key factor in the development of resistance. To investigate this issue, we selected two glycol porphyrin derivatives with diverse intracellular localization, KP1 and KP6, which were prepared in our laboratory as compound **2** and compound **6,** respectively^18^. These two particular derivatives were specifically chosen for the study of PDT resistance mechanisms because as neutral and highly water-soluble glycol porphyrin derivatives are known to be very potent inducers of apoptosis in tumor cells and exhibit a high potential for *in vivo* PDT applications. Moreover, in spite of their chemical structural similarity, they are varied in both intracellular localization and apoptosis-triggering mechanism^18^,^19^. The differences between the KP1 and KP6 derivatives can be found in the position of their ethylene glycol chains which are linked *via* ether bonds to either meta- or para-phenyl positions of meso-tetraphenylporphyrin, respectively. In addition, the para-derivative, KP6, is fluorinated to enhance its photosensitizing potential^19^. Temoporfin, meta-tetra(hydroxyphenyl)chlorine (mTHPC), which is used in PDT of squamous cell carcinoma of head and neck^20^ under the brand name Foscan, is a member of a different family of PS and was included in our study to illustrate the variety of resistance mechanisms. The results obtained in this study indicate that the chemical structure of PS rather than a particular intracellular localization is the determining mechanism of PDT resistance. PS with a similar structures adopted similar resistance mechanism(s) employing upregulated ABC transporters, while the structural difference of temoporfin, in spite of analogous intracellular localization, led to more diverse mechanism(s).

## Results

### Derivation and characterization of PDT-resistant cancer cells

**Figure 1:**
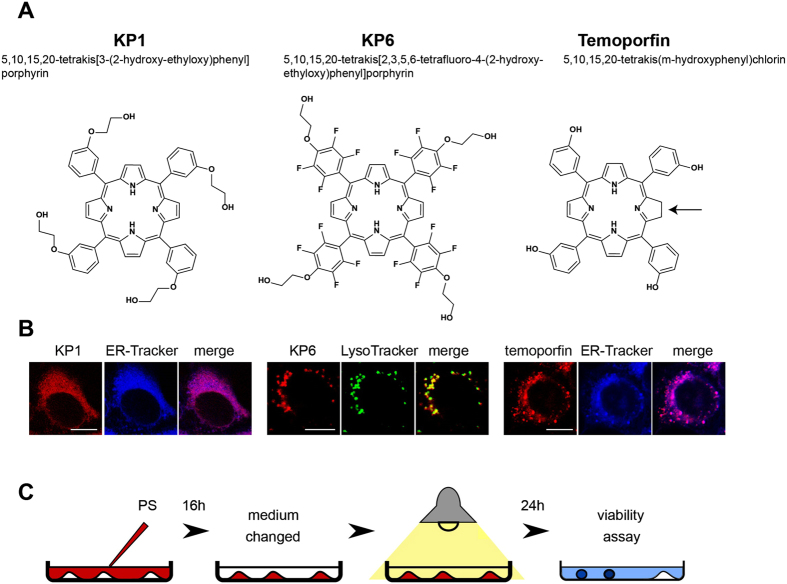
Structures, intracellular localization and experimental applications of used PS. (**A**) Structures, elemental analysis and synthesis of glycol porphyrin derivatives KP1 and KP6 were published before^18^. Temoporfin heteroaromatic ring, in contrast to porphyrin, consists of three pyrrols and one pyrroline subunit pointed by arrow. (**B**) Co-localization of KP1 and temoporfin fluorescence (red) with ER-Tracker (blue) merged into pink color, while KP6 accumulation in lysosomes with LysoTracker (green) merged into yellow in 4T1 cells. Scale bar represents 10 μm. (**C**) The general scheme of experimental PDT application. For specification of used concentrations and light exposure, see Supplementary Table S1.

**Figure 2:**
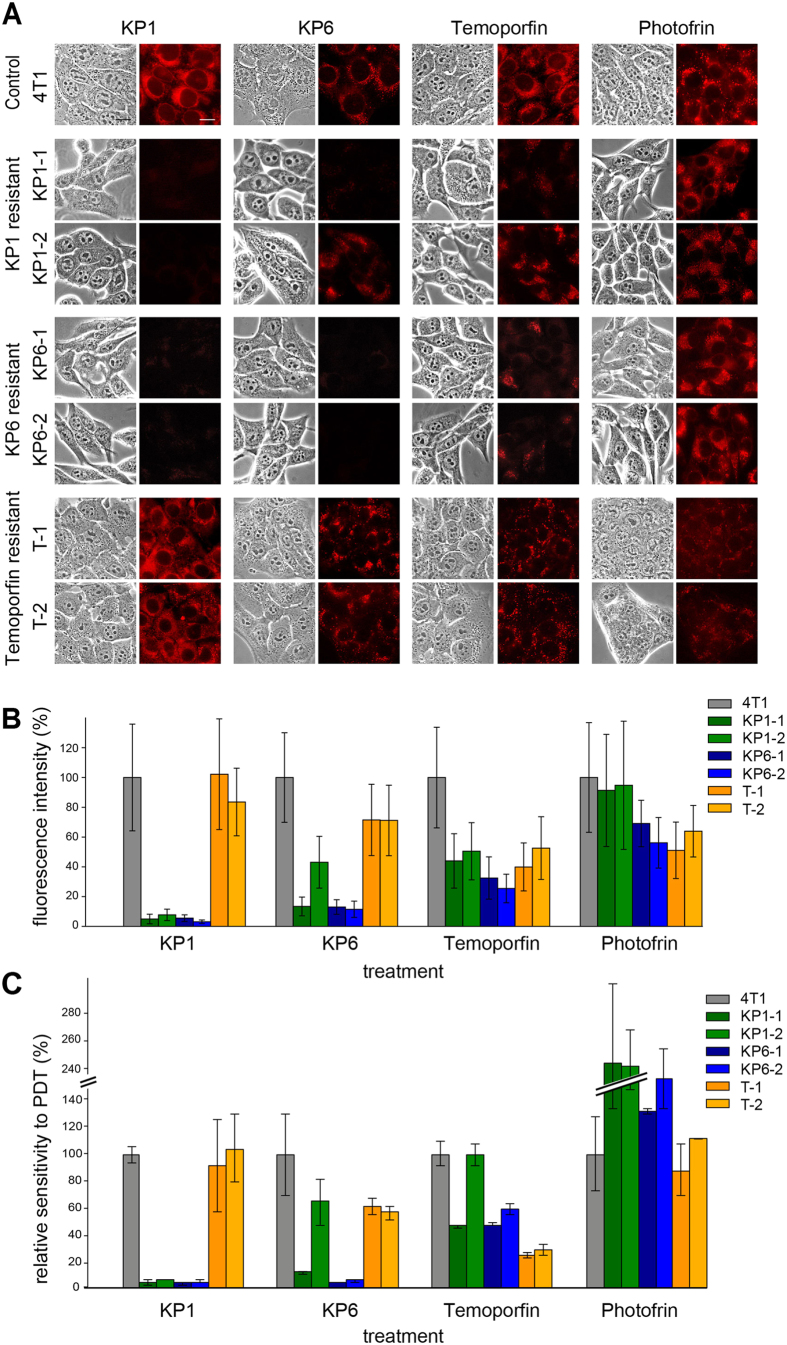
Characterization of PDT-resistant cells. (**A**) Fluorescence of PS in parental cells 4T1 and clones with acquired resistance. Cells (in rows) were incubated with KP1, KP6, temoporfin and photofrin (in columns) and inspected under a Leica microscope. The phase-contrast images are shown on the left side and PS fluorescence images of the same field on the right side of each column. Scale bar represents 10 μm. (**B**) Quantitative evaluation of PS fluorescence intensity. Graph summarizes relative fluorescence intensities of resistant cells taken under identical camera setting from different fields relative to parental cells. (**C**) Relative sensitivity to PDT. Resistant cells were loaded with PS and exposed to various doses of light to establish LD50. Relative sensitivity to PDT (%) reflects the difference in responsiveness of a particular clone relatively to parental cells 4T1.

The list of PS used in this study for inducing resistance which includes their structure, intracellular localization and experimental application is summarized in Fig. 1. To establish cell lines that are resistant to PDT, we subjected 4T1 mouse mammary carcinoma cells to the selection procedure specified in Supplementary Table S1. Specific conditions such as PS concentration and light wavelength, power density as well as light dose were chosen for each PS individually in such a way that the PS was not toxic by itself but it induced 90% lethality after irradiation. Ten to twenty cycles of selection were necessary to achieve resistance. Two clones were isolated from the same selection cycle of PDT by limiting dilution and further analyzed. First, the cells were incubated with the primary PS which was used for selection and a cross-challenging PS and the intrinsic fluorescence of the PS was observed (Fig. 2A). Porfimer sodium, a mixture of porphyrin oligomers known under the brand name Photofrin commonly used in the treatment of esophageal and endobronchial cancers^21^, was also included as a cross-challenging agent to assess the level of cross-resistance. The fluorescence of KP1 and KP6 was strongly reduced in both KP1- and KP6-resistant clones (Fig. 2A,B). Interestingly, clones resistant to KP1 and KP6 did not display such consistent reduction of fluorescence intensity following photofrin challenge. Cross-challenge of the same clones with temoporfin resulted in attenuated fluorescence. Temoporfin-resistant clones, in contrast to KP1 and KP6, displayed reduced but still substantial fluorescence compared to temoporfin and the other cross-challenging PS. The intracellular concentrations of PS were quantitatively determined in cell lysates of resistant clones by measuring their fluorescence emission spectra (see Supplementary Fig. S1) which revealed similar differences observed in epi-fluorescence microscopy examination (Fig. 2).

The sensitivity to PDT was established for each clone in terms of LD_50_, which is the light dose that is required to kill 50% of the cells at given concentrations of PS (for actual data see Supplementary Table S2). In addition, the relative sensitivity which represents the sensitivity of a particular clone in contrast to parental cells 4T1 (Fig. 2C), and the resistance index (RI), defined as LD_50_ of resistant clone/LD_50_ of parental cells, can be used as another type of expression for acquired resistance. The RI is listed in Supplementary Table S2.

The relative sensitivity to PDT with various compounds showed a clear correlation with fluorescence intensity of the cells. When treated with KP1, the clones selected for resistance to either KP1 or KP6 displayed very low sensitivity, while clones selected for temoporfin resistance exhibited similar sensitivity as the control (Fig. 2C - seven left bars). Upon KP6 treatment, both KP6-resistant clones and one KP1-resistant clone showed very low sensitivity, while the other KP1-2 resistant clone retained its high sensitivity. Interestingly, both temoporfin-resistant clones were also markedly less sensitive to KP6 than the control. Sensitivity to temoporfin was relatively high in all clones. It is of note that even those that were selected for temoporfin resistance retained partial sensitivity (~25%). Other clones were less sensitive in comparison to the control with the exception of KP1-2. Interestingly, both KP1-, and to a lesser extent KP6-resistant clones, exhibited increased sensitivity to photofrin, while temoporfin-resistant cells showed no change.

### *In vivo* tumorigenicity of PDT-resistant clones

**Figure 3:**
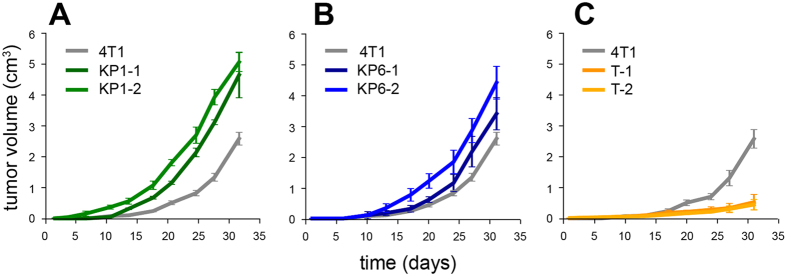
*In vivo* tumorigenicity of PDT-resistant clones in BALB/c mice. Control cells (4T1) and cells of PDT-resistant clones were injected subcutaneously into syngeneic mice BALB/c to assay their growth potential *in vivo*. Tumor growth was evaluated by measuring tumor dimensions by calipers every third or fourth day. **(A–C)** The average of tumor volumes originated from cells of KP1-, KP6- and temoporfin-resistant clones, respectively, were plotted against time and compared to parental cells. Each group consist of four animals.

The ability of selected PDT-resistant cell line clones to form tumors was evaluated in the syngeneic mouse strain, BALB/c. The measurable tumors formed by KP1- and KP6-resistant cells appeared 8–10 days after subcutaneous inoculation, similarly to parental 4T1 cells. By day 15, however, the rapid growth of the tumors had been recorded and by day 30, the tumors had already reached a significantly larger size than those found in the control parental group (Fig. 3A,B). In contrast, the ability of temoporfin-derived PDT-resistant clones to form tumors *in vivo* was considerably decreased (Fig. 3C) despite their similar growth potential *in vitro* shown by cumulative cell growth curves, [H3] thymidine incorporation and doubling time (see Supplementary Fig. S2).

### Expression profiling

**Figure 4:**
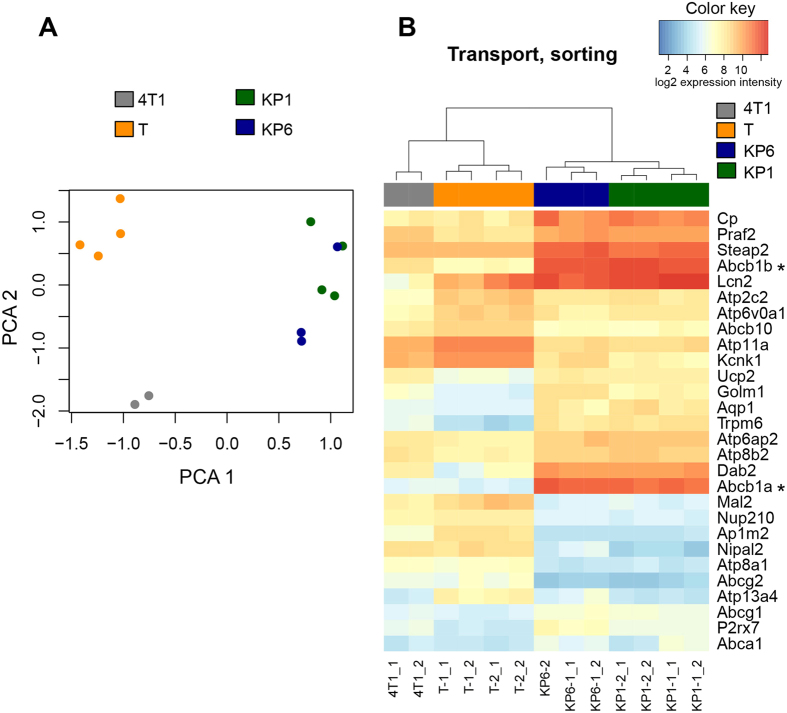
Microarray analysis identified marked differences between the expression profiles of PDT-resistant cells and parental cells. (**A**) Principal component analysis (PCA) of the whole-genome expression data indicates close resemblance of the gene expression profile of KP1 and KP6 clones unlike temoporfin (T) resistant clones. (**B**) Heatmap of the expression intensities of selected genes associated with cellular transport and sorting. Asterisks point at strong upregulation of *Abcb1* genes. For other deregulated clusters of functionally related genes, see Supplementary Fig. S1 and Supplementary Table S3.

To grasp mechanisms which might be responsible for the development of resistance against different PS, microarray analysis of mRNA isolated from KP1-, KP6- and temoporfin-resistant clones was performed and compared with parental 4T1 cells. Each selected resistance group (KP1, KP6, temoporfin) was represented by two clones, which were analyzed in duplicate from separate RNA isolations. Untreated control parental cells served as the baseline for sample comparisons. Multiple upregulated and downregulated genes were found in each resistance group. The profile of different isolates of each clone and representative clones of each group were mostly consistent, with just a few exceptions, and generally did not show substantial disparity (Fig. 4A). The genes that showed significantly changed expression in PDT-resistant clones versus parental 4T1 cells are involved in many different cellular processes and can be grouped into specific functional classes (see Supplementary Fig. S3, Supplementary Table S3).

Despite the fact that some genes showed upregulation in all PDT-resistant groups (*Txnip, Lcn2, Ptgs1*, and *Nos2*; see Supplementary Table S3), most of the genes displayed differential expression depending on the resistance group. Generally, the expression profile of KP1 and KP6 clones was similar and included the same genes with similar rates of change, however, one notable exception was the detection of upregulation of *Gstk1* in KP6 and not in KP1 clones (see Supplementary Table S3). Consistently, a striking upregulation of ATP-binding cassette transporter *Abcb1a* and *Abcb1b* was found in KP1 and KP6 clones (Fig. 4B, Supplementary Table S3). In contrast to KP1 and KP6, temoporfin-resistant clones did not show substantial changes in ABC transporters but displayed significant changes in many other genes (Fig. 4, Supplementary Fig. S3, Supplementary Table S3). The differences found in fluorescence, sensitivity to PDT, tumorigenicity and gene expression profile between the KP1 and KP6 groups and the temoporfin group indicated different mechanisms of resistance, which we further analyzed.

### Potential mechanisms which play a role in temoporfin resistance

**Figure 5:**
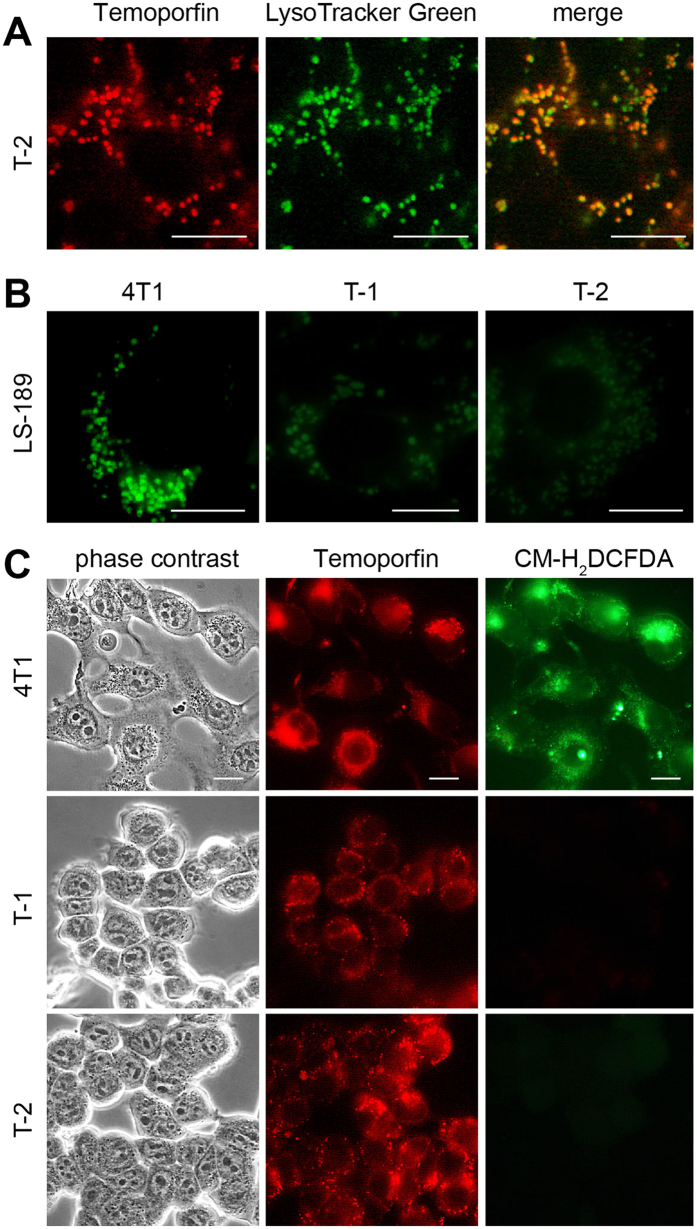
Specifics of temoporfin-resistant clones. (**A**) Temoporfin relocalizes to lysosomes in temoporfin-resistant clones. Cells of the T-2 clone were incubated with temoporfin (1 μM) and then subsequently for 30 min with LysoTracker probe (0.5 μM). (**B**) The fluorescence of pH-sensitive probe LysoSensor ™ Green 189 (3 μM, 30 min incubation) is strongly reduced in temoporfin-resistant clones in contrast to parental 4T1 cells. (**C**) ROS-specific probe CM-H_2_DCFDA does not detect the ROS formation after light exposure in temoporfin-loaded resistant clones. For comparison with other resistant clones, see Supplementary Fig. S2. Scale bar represents 10 μm.

Primarily, the subcellular distribution of temoporfin was changed from the prevailing ER localization in parental cells (Figs 1B and 2A) to predominantly lysosomal localization in temoporfin-resistant clones, as detected by the lysosomal probe, LysoTracker Green (Fig. 5A). In addition, the pH in the lysosomal compartment of temoporfin-resistant clones became more alkaline as indicated by the reduced fluorescence of the pH-specific probe, LysoSensor LS-189 (Fig. 5B), whose pKa is 5.2. LS-189 provided bright fluorescence in parental cells, KP1- and KP6-resistant cells but not in temoporfin-resistant clones. This demonstrates that the pH increase is specific only for the lysosomal compartment of temoporfin-resistant clones. In addition, temoporfin photoactivation by light in these clones did not result in the formation of reactive oxygen species (ROS), detectable by the specific probe CM-H_2_DCFDA. In contrast, temoporfin-loaded parental cells (Fig. 5C) and KP1 and KP6 clones provided a positive ROS signal (see Supplementary Fig. S4).

It is also noteworthy that remarkable changes in cytoskeletal genes (upregulation of *Tubg2*, downregulation of *Vim*), genes involved in cell adhesion (downregulation of *Vcam*, upregulation of *Gpr87*), the integrin pathway (upregulation of *Cldn 6, Tspan 1, Dok2*), metabolism (upregulation of *Aspa, Cers3*), and genes of the *Ras* superfamily (downregulation of *Rgs16, Gnb4*) along with many others were identified in temoporfin-resistant clones (Supplementary Table S3). Changes in mRNA expression were also validated at the protein level for some targets, as exemplified by vimentin and ceramide synthase 3 (see Supplementary Fig. S5).

### Mechanisms of KP1 and KP6 resistance to PDT

**Figure 6:**
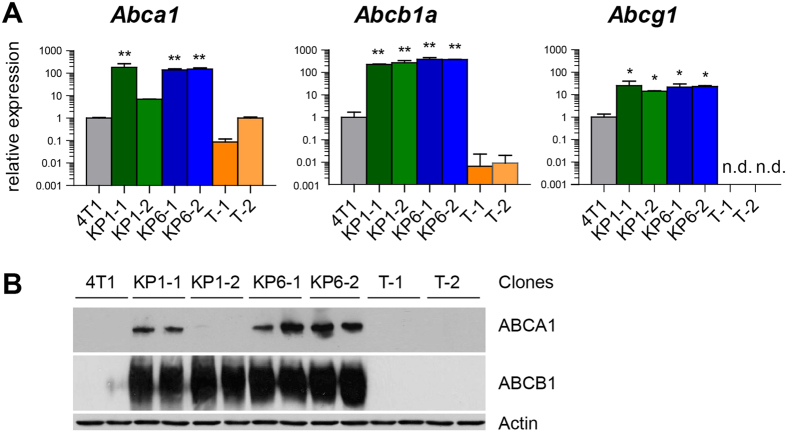
Expression of ABC transporters in PDT-resistant clones. (**A**) qPCR analysis of most significantly upregulated ABC transporters. Expression of individual murine ABC transporters was measured using the TaqMan^®^ Array Mouse ABC Transporters from Life Technologies. *Gapdh* was used for normalization. Each clone was analyzed in biological duplicate. Stars denote the significance of difference from 4T1 cells; one star, p < 0.05, two stars, p < 0.01. For actual data see Supplementary Table S4. (**B**) Western blot analysis of the most upregulated ABC transporters. The membrane carrying blotted proteins from cellular extracts of parental and PDT-resistant clones in biological duplicates was sequentially probed with ABCA1, ABCB1 antibodies and eventually with actin antibody to demonstrate equal protein loading.

Microarray analysis revealed strong upregulation of *Abcb1* mRNA in KP1- and KP6-resistant clones, therefore ABC transporter-associated genes became the focus of our analysis. We implemented TaqMan Array qPCR covering all seven ABC transporter subfamilies of mouse origin. The results were in line with the microarray data showing strong upregulation of *Abcb1a* (Fig. 6A, Supplementary Table S4) in KP1- and KP6-resistant clones. *Abcb1b* was not measured as it was not part of the TaqMan mouse kit. In addition, elevated *Abca1* expression, which was not present in all clones (missing in KP1-2), and *Abcg1* expression was detected (Fig. 6A). Interestingly, the mRNA expression of *Abcg2* in KP1- and KP6-resistant clones was downregulated in a similar manner as seen in the microarray data (Supplementary Tables S3 and S4). The profound change in the ABCB1 expression was validated at the protein level by Western blot analysis (Fig. 6B). Elevated ABCA1 expression was observed in KP6 and KP1-1 clones but was not detected in the KP1-2 clone (Fig. 6B). The detection of ABCG1 protein using a polyclonal antibody was not reproducible (data not shown), and thus mRNA data remain unconfirmed.

### Overexpression of ABCB1 in paclitaxel-resistant cells imparts resistance to KP1 and KP6-mediated PDT

**Figure 7:**
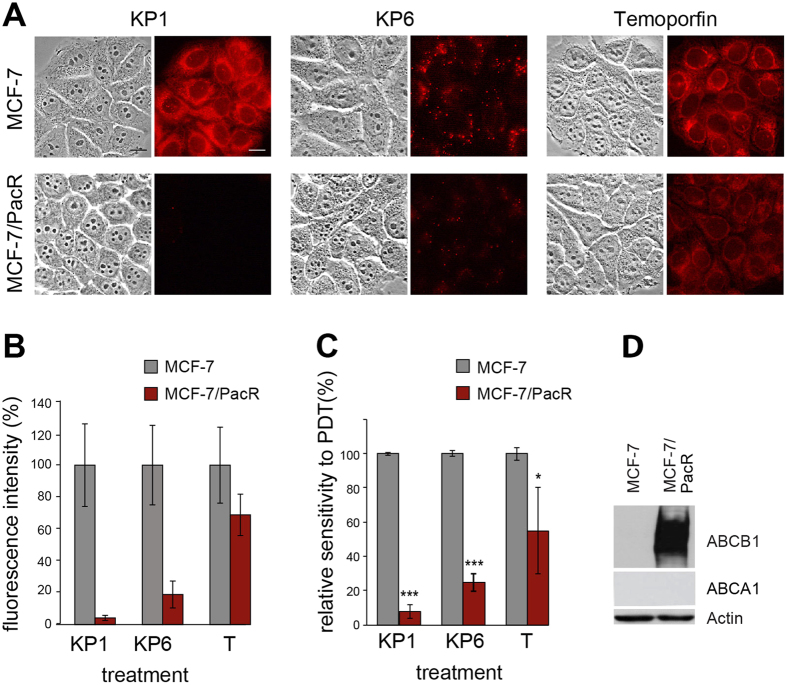
Overexpression of ABCB1 in paclitaxel-resistant cell decreases fluorescence and imparts resistance to KP1 and KP6. (**A**) Paclitaxel-resistant cells with high expression of ABCB1 transporter^22^,^48^ were tested for their ability to accumulate KP1, KP6 and temoporfin (T). The fluorescence of PS (shown in columns) in paclitaxel-sensitive MCF-7 and paclitaxel-resistant MCF-7/PacR cells (shown in rows) was monitored by fluorescence microscopy. Reduced accumulation of KP1 and KP6 as measured from the images (**B**) strongly correlates with sensitivity of MCF-7/PacR cells to PDT (**C**). The graphs represent the mean of at least three experiments with standard deviation. Stars denote the significance of difference from MCF-7 cells; one star, p < 0.05, three stars, p < 0.001. (**D**) Confirmation of strong expression of ABCB1 in MCF-7/PacR cells by Western blot.

To verify the causal link between ABCB1 overexpression and the origin of PDT resistance derived from KP1 and KP6 porphyrins, we investigated cells with acquired MDR resistance to paclitaxel strongly overexpressing ABCB1^22^,^23^. Paclitaxel-resistant cells MCF-7/PacR were loaded with PS and their fluorescence and phototoxicity after light exposure was evaluated and compared to parental paclitaxel-sensitive MCF-7 cells. The fluorescence pattern of PS in MCF-7 cells was very similar to 4T1 (top panel in Fig. 2A and Fig. 7A). In MCF-7/PacR, however, the fluorescence of KP1 and KP6 was substantially reduced, although to a slightly lower degree in the case of KP6 (Fig. 7A,B). Accordingly, the PDT sensitivity varied depending on the PS used (Fig. 7C and Supplementary Table S5). The MCF-7/PacR cells exhibited low sensitivity to KP1 (only about 8% in comparison to parental MCF-7 cells), which indicates a major involvement of ABCB1 in the KP1 efflux leading to PDT resistance. A less significant decrease of sensitivity was found after KP6 (about 25%) and temoporfin challenge (55%).

### Knockdown of ABCB1 rescues the sensitivity to PDT

**Figure 8:**
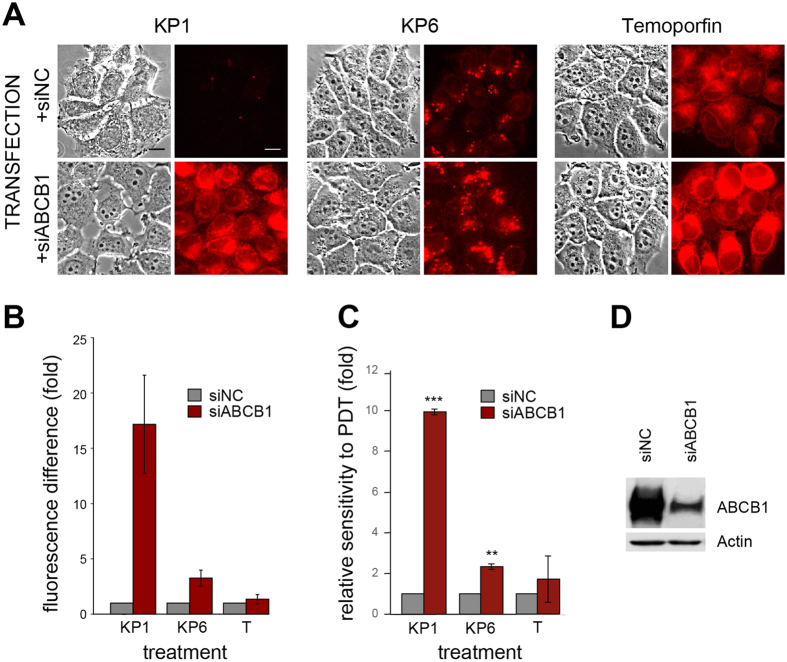
siRNA knockdown of ABCB1 leads to accumulation of KP1 and KP6 and restoration of sensitivity to PDT in MCF-7/PacR cells. MCF-7/PacR cells were transfected with siRNA targeting ABCB1 (+siABCB1) or with non-targeting negative control siRNA (+siNC) and challenged after 72 h with KP1, KP6 and temoporfin (T). (**A**) The fluorescence following PS treatment was monitored under a fluorescence microscope; pictures were taken under identical camera settings. The phase-contrast images are shown on the left side and PS fluorescence images of the same field on the right side of each column. Scale bar represents 10 μm. siRNA targeting of ABCB1 resulted in a significant increase of PS fluorescence (**B**), which correlated with an increase of relative PDT sensitivity defined as fold change of LD50 of +siNC-transfected cells/LD50 of +siABCB1-transfected cells (**C**). Suppression of the ABCB1 protein was confirmed by Western blot (**D**).

siRNA knockdown of ABCB1 expression in MCF-7/PacR cells provided another piece of evidence demonstrating that ABCB1 upregulation is a relevant cause of KP1 and KP6 acquired PDT resistance. The strong expression of ABCB1 in MCF-7/PacR cells was markedly suppressed by ABCB1 siRNA transfection but not by non-targeting siRNA (siNC) which was used as a control (Fig. 8D). When these transfected cells were incubated with PS, they displayed differential fluorescence (Fig. 8A). The most dramatic increase of fluorescence intensity in cells transfected with siABCB1 in comparison to siNC was observed upon KP1 challenge (~16 times over the control). KP6 challenge resulted in a 3-fold increase in fluorescence, while temoporfin showed only a marginal increase. The fluorescence intensities of KP1 and KP6 correlated well with the relative sensitivity toward KP1- and KP6-mediated PDT, showing about a 10-fold increase of sensitivity to KP1 and 2.7-fold to KP6. Thus, these siRNA experiments point out that the ABCB1 transporter plays a key role in the efflux of KP1 and partially KP6 from these cells, thereby imparting PDT resistance toward these PS.

## Discussion

Photosensitizers are critical elements in PDT. Although the quantity and location of PS can, to a certain extent, predict the nature of photodynamic reactions and determine the consequences of the anticancer effect, it is evident that at equivalent cellular levels of PS, there are other factors that might impact the cellular sensitivity as well as phototoxicity. Our cellular models of resistance using closely related porphyrin ethylene glycol derivatives and temoporfin provided new insights into the molecular basis of resistance to PDT.

### Resistance to temoporfin is associated with its lysosomal sequestration and changed lysosomal metabolism

Our study confirmed the general consensus that the chemical structure of PS is a key element in the mechanism of resistance to PDT. Temoporfin contains the chlorin core which, unlike the porphyrin central aromatic ring, consists of three pyrroles and one pyrroline coupled through four = CH- linkages. Therefore, temoporfin is not aromatic throughout the entire circumference of the ring^24^. This structural difference leads to changes in physicochemical properties and in the spectral characteristics^25^,^26^. Although the conclusions from existing literature data questioned the possibility of inducing resistance to temoporfin-mediated PDT^27^, we succeeded in creating resistant cells *via* multiple selection cycles. The temoporfin-resistant clones differed from porphyrin derivatives in some key characteristics. Specifically, the relocalization of temoporfin to lysosomes (Fig. 5A,B) and more alkaline milieu of the lysosomes as indicated by pH-sensitive probes (Fig. 5B) were observed in temoporfin-resistant cells. We assumed that the lower protonation of temoporfin due to alkalization of lysosomal vesicles resulted in temoporfin aggregation and subsequently inferior fluorescence (Fig. 2A,B) as well as a drop in PDT effectivity (Fig. 2C). This notion was supported by the poor formation of cytotoxic ROS, which are the primary inducers of cell death after light irradiation (Fig. 5C, Supplementary Fig. S4). An alternative explanation based on the increased inactivation of ROS via detoxifying enzymes and activation of heat-shock proteins^2^ seems to be less likely since no changes in the expression profile of these proteins were found in temoporfin-resistant cells, although they may play a role at the posttranscriptional level. Yet, the molecular mechanism of the change in lysosomal milieu is not clear. A detailed search of microarray data in temoporfin-resistant clones revealed moderately downregulated *Atp6ap2*, which is a regulator of vacuolar proton ATPase, the protein complex which is responsible, among others, for lysosomal acidity^28^. This could explain the change of the lysosomal milieu in resistant cells but additional studies are needed to confirm this. The proposed mechanism of lysosomal sequestration does not exclude the possibility of other factors that may have a role in the temoporfin resistance.

Another clue suggesting a role of lysosomal metabolism in temoporfin resistance comes from the study of cross-resistance. Temoporfin-resistant clones retained sensitivity to KP1 and photofrin, whereas they became partially resistant to KP6 (Fig. 2C, orange bars). We believe that this resistance to KP6 is caused by the natural localization of KP6 in lysosomes (Fig. 1B). While lysosomal KP6 in parental cells is able to cause considerable phototoxicity, in temoporfin-resistant clones with changed lysosomal milieu KP6 is less effective. In contrast, KP1, which is localized in ER in both parental and temoporfin-resistant cells, elicits strong phototoxicity (Fig. 2A,C).

### Changes in cytoskeleton and cell adhesion

The microarray analysis revealed significant changes in the expression of genes encoding proteins that are associated with the cytoskeleton, cell adhesion (including several components of the integrin pathway), metabolism, and many others in all resistant clones (Supplementary Table S3), which visibly altered cell shape (Fig. 2A) and adhesion behavior. Specifically, temoporfin-resistant clones displayed significant downregulation of a major cytoskeletal protein, vimentin (Supplementary Fig. S3, Supplementary Table S3), and upregulation of γ-tubulin 2. Recent studies have linked the changes in cytoskeleton (actin, α-tubulin) and cellular adhesion mediators (E-cadherin, integrins, fibronectin) to the development of PDT resistance^3^,^29^ and metastatic potential of tumor cells^30^. In animal models, PDT was shown to affect the metastatic potential of various tumors^31^,^32^. Interestingly, photosensitization with phthalocyanine Pc4 was linked to vimentin cleavage which precedes poly(ADP-ribose) polymerase (PARP) degradation, a characteristic sign of apoptosis^33^. It is possible that downregulation of vimentin helps cells to avoid this apoptotic pathway and thus facilitates development of resistance to temoporfin. Temoporfin-resistant clones were also more refractory to trypsinization and showed a high tendency to remain as aggregates. However, the changes in the gene expression which is responsible for these effects and their relevance to the mechanism of resistance remain elusive.

### Effect of ABC transporters on KP1 and KP6 resistance

The investigation of KP1 and KP6 resistance showed that the porphyrin structural motif with ethylene glycol substitution on the periphery determines both derivatives as substrates for MDR transport and efflux mediated by ABC transporters. This conclusion, which is in line with the observations made for other PS^16^, is supported by several lines of evidence. Both KP1 and KP6 PDT-resistant clones were characterized by markedly elevated levels of both *Abcb1* transcript (Figs 4 and 6A) and protein (Fig. 6B). Accordingly, resistant cells displayed reduced fluorescence and phototoxicity after KP1 and KP6 exposure (Fig. 2). Moreover, when resistant clones were exposed to the same light dose but increasing doses of KP1 and KP6, the sensitivity to PDT was restored (see Supplementary Fig. S6). This observation demonstrates that high concentrations of PS can saturate the drug pump, which validates the efflux-driven mechanism. The actual doses of PS required for reaching IC80 in KP1-resistant clones were 10–14 times higher than parental cells while only 4–5 times higher in KP6 clones. When these cells were pre-treated with transporter inhibitors (cyclosporin A, probenecid, MK-571), although their specificity was slightly questionable, the fluorescence of KP1 and KP6 as well as the sensitivity toward PDT was, at least in part, increased (Supplementary Fig. S7). Moreover, KP1- and KP6-resistant clones displayed substantial cross-resistance when challenged with KP6 and KP1, respectively (Fig. 2, Supplementary Table S2), hence demonstrating similar mechanisms of resistance.

However, the assessment of cross-resistance indicated a plausible participation of another transporter, ABCA1, whose selective expression was shown by qPCR and Western blot analysis (Fig. 6, Supplementary Table S4). In addition, the possible differential affinity of the transporters to KP1 and KP6 substrates as well as the duration of the selection process might also play a role. For example, KP1 clones expressed a consistently high level of ABCB1 but differed in the levels of ABCA1. Upregulation of ABCA1 was detected in the KP1-1 clone and correlated with the appearance of cross-resistance to KP6, while the KP1-2 clone neither expressed ABCA1 nor was fully resistant to the KP6 challenge (Figs 2 and 6). We hypothesize that cells with upregulated ABCB1 are enriched early during selection due to its high affinity to both KP1 and KP6. Later, with increasing selective pressure (higher light doses), ABCA1 upregulation becomes more important. The establishment of cells resistant to KP1-mediated PDT required fewer additional cycles compared to KP6-resistant cells (Supplementary Table S1), thus isolation of KP1 clones could have occurred at the stage of an increasing but not yet fixed level of ABCA1 transporter in the mixed cell population, which resulted in differences between the clones. In contrast, numerous selection cycles before the isolation of KP6 clones facilitated fixation of ABCA1 expression in the entire population, and thereby expressed in both analyzed clones. Moreover, a potential difference in the affinity of ABCA1 to KP6 and KP1 substrates may be an another reason for the more prominent role of this transporter in KP6 resistance. In this respect, we observed, interestingly, strong upregulation of ABCB1 but not ABCA1 (Fig. 7D) in paclitaxel-resistant cells. These cells were still partially sensitive to KP6-PDT (Fig. 7C), showing that ABCB1 itself was not sufficient to mediate complete drug efflux and cross-resistance (Fig. 7, Supplementary Table S5). Taking into account many other specific factors that affect PDT and paclitaxel resistance, the involvement of ABCA1, particularly in KP6 resistance, is highly probable. To the best of our knowledge, the only published data indicating the elevation of ABCA1 after hypericin-mediated PDT treatment has not sparked much interest^34^. Interestingly, KP1- and KP6-resistant clones were more sensitive to photofrin-mediated PDT than parental cells (Fig. 2C). Photofrin was recognized as a substrate of the ABCG2 transporter in several reports^35^,^36^,^37^, therefore downregulation of *Abcg2* in KP1 and KP6 clones could make these cells more sensitive to photofrin-mediated PDT relatively to parental cells (Fig. 4, Supplementary Table S3). We have provided convincing data *via* experiments with siRNA-mediated specific knockdown in MCF-7/PacR cells overexpressing ABCB1 that the ABCB1 transporter is responsible for the development of resistance to KP1 and KP6. The cells displayed rescued sensitivity, as revealed by increased fluorescence and relative sensitivity to PDT (Fig. 8). The level of sensitivity differed depending on PS. While reduction of ABCB1 level resulted in the restoration of sensitivity toward KP1, only partial rescue of sensitivity was achieved from the KP6 challenge, suggesting the involvement of ABCA1 in KP6 efflux which was discussed above. To the best of our knowledge, this is the most conclusive demonstration of ABCB1 involvement in PDT resistance induced by porphyrins. In spite of the fact that the overall overexpression of ABCB1 and ABCA1 was identified as the main cause of KP1- and KP6-mediated PDT resistance, the contribution of other factors cannot be ruled out.

### The impact of solubility on PS performance and susceptibility to resistance

A major problem with PDT efficacy is generally the low solubility of PS, which consequently leads to aggregation. In some cases, aggregation merely diminishes the activity of the PS, whereas with others it may change its mechanisms of action^38^. The tetra-ethylene glycol derivatives were originally prepared in our laboratory to improve solubility^18^,^19^. The improvement in solubility might be the reason for their higher PDT efficacy in comparison to temoporfin. However, the monomeric form of glycol porphyrins could be more susceptible to efflux by transporters while more lipophilic temoporfin is not effectively pump out.

### Summary

Our cellular model revealed different molecular mechanisms eliciting PDT resistance to porphyrin derivatives and temoporfin. We found that the resistant cell line variants had distinctive temoporfin- or porphyrin-specific resistance profiles, with nearly non-reciprocal cross-resistance. In temoporfin resistance, the sequestration of PS to lysosomes and their alkalinisation, which results in aggregation, poor ROS formation and low photodynamic efficacy, seems to play a major role. Both KP1 and KP6 porphyrin derivatives, in spite of their different intracellular localization and mechanism of action, become targets of the ABCB1 transporter. The possible involvement of another transporter, ABCA1, is also supported, mainly in the context of KP6 resistance. Furthermore, it was found that PS eliciting resistance by similar mechanisms create cross-resistance, which could be circumvented by drugs that tend to be eliminated differently. Thus, temoporfin resistance can be reverted by using KP1 and photofrin, while KP1 and KP6 resistance can be overcome by photofrin and partly by temoporfin. In addition, several new, along with some previously described, drug resistance-associated genes were identified in each resistant cell line variant. Each PDT resistant cell line variant acquired a unique set of changes that may represent distinct functional subtypes of PDT therapy resistance. Based on these results, we believe that our data contributes to a better understanding of PDT resistance mechanisms, which could in turn lead to more rational design of strategies combining PDT with chemotherapy or other modalities in the treatment of cancer.

## Material and Methods

### Photosensitizers

Synthesis and elemental analysis of the porphyrin derivatives KP1 (meta-ethyleneglycol-tetraphenyl porphyrin), KP6 (para-ethyleneglycol-tetraperfluorophenyl porphyrin) and temoporfin (meta-hydroxy-tetraphenyl chlorin) was performed at the University of Chemistry and Technology, Prague and have been described elsewhere^18^. Photofrin (Axcan Pharma International, Sittard, NL) was used as a control.

### Chemicals

The monoclonal antibody against vimentin, VI-01, was a kind gift from Dr. Draberova (IMG, Prague, CZ)^39^. Anti-actin antibody (A2066) was purchased from Sigma-Aldrich (St. Louis, MO, USA). Rabbit monoclonal anti-P-glycoprotein (ab170904) and ceramide3 synthase-Anti-LASS3 polyclonal antibody (ab28637) were obtained from Abcam (Cambridge, UK), Abca1 (mAB10005) from Merck Milllipore Corporation (Darmstadt, DE) and Abcg1 (PA1-16804) from Thermo Fisher Scientific (Waltham, MA, USA). Transport inhibitors probenicid and cyclosporin A were obtained from Sigma-Aldrich, MK-571 from Enzo Lifescience (Farmingdale, NY, USA). Molecular probes ER-Tracker™ Blue-White DPX, LysoSensor™ Green DND-189, LysoTracker^®^ Green DND-26, and the general oxidative stress indicator CM-H_2_DCFDA were purchased from Thermo Fisher Scientific. Paclitaxel was purchased from Sigma-Aldrich.

### Cell lines and cell culture

Mouse mammary carcinoma 4T1 and human breast adenocarcinoma MCF-7 were obtained from ATCC (Manassas, VA, USA). Cell lines were maintained at exponential growth in RPMI 1640 medium with specific supplements used for 4T1^40^ and for MCF-7^41^ at 37 °C in a humidified 5% CO_2_ atmosphere. Paclitaxel-resistant MCF-7 cells (MCF-7/PacR) were prepared in the laboratory of Prof. Jan Kovář (Third Faculty of Medicine, Charles University in Prague) by gradual adaptation of the original taxane-sensitive cell line to increasing paclitaxel concentration up to 300 nM paclitaxel in culture medium^22^. Medium supplemented with DMSO-dissolved paclitaxel (300 nM) was prepared fresh weekly. Paclitaxel in the medium was omitted during siRNA experiments.

### Photodynamic treatment

Cells (2–3 × 10^5^) were seeded in 35 mm dishes and left to grow for one day at 37 °C in 2 mL of RPMI media. Cells were then incubated for 16 h with KP1 (0.7 μM), KP6 (1.6 μM), or temoporfin (1 μM) and kept in the dark. All cell manipulations were performed under subdued light conditions. The cells were rinsed with PBS, overlaid with fresh medium without phenol red and then exposed to light. For all photo-activation experiments with porphyrins KP1 and KP6, an illuminator equipped with a 75 W halogen lamp and a bandpass filter 500FS40-50 (Andover, Salem, NH) was used. The power density at the level of cell monolayer was 0.7 mWcm^−2^ and the total light dose ranged in 0.5–13 J cm^−2^. For temoporfin and photofrin, a bandpass filter 650FS80-25 transmitting light with power density 3.7 mW cm^−2^ was used and the total light dose ranged from 1 to 26 J cm^−2^. On the day following irradiation, the viability of post-PDT cultures was determined by the Trypan blue exclusion. Control “dark” experiments were performed in parallel with only the irradiation step omitted.

### Selection of PDT-resistant cells

4T1 cells were treated with KP1, KP6 and temoporfin in conditions (concentration and light dose) that promoted survival of only 1–10% cells. The cells from each cycle were harvested, plated in a new dish, and their resistance was verified. Subsequently, cells were subjected to new cycles of PDT treatment using higher light dose. Individual clones were isolated from selected cell populations by limiting dilution. The sensitivity to PDT was established for each clone in terms of LD_50_ value, a light dose which leads to the killing 50% of the cells at a given concentration of PS, and the relative sensitivity expresses the sensitivity of a particular clone in contrast to parental 4T1 cells in percentage. Alternatively, as another type of sensitivity expression we used the resistance index (RI), defined as LD_50_ of the resistant clone/LD_50_ of parental cells.

### Proliferation assays

Cell proliferation was measured using thymidine incorporation assays. Cells were plated at a density of 10^4^ cells/well in 96-well plates. After 12 hours, cells were pulsed with [methyl-^3^H] thymidine (UJV Rez) and cultured for an additional 2 hours. Cells were harvested onto a Filtermat using a FilterMate Harvester. Incorporated radioactivity was quantified using a MicroBeta2 Microplate scintillation counter (PerkinElmer).

A cumulative growth curve was established by reseeding cells at a density of 2.5 × 10^4^/cm^2^ every other day and counting the number of cells using a hemocytometer.

Generation time or doubling time (G) is the time required for duplication of cells in the culture. It was calculated according to the formula G = t.log2/log N_t_ − log N_0_, where t signifies the time of growth, N_0_ refers to plating number of cells, and N_t_ number of cells obtained at the time of harvest.

### Fluorescence microscopy

Cells grown on coverslips in 35-mm Petri dishes were incubated with the corresponding PS in complete medium for 16 h, with the exception of photofrin which was exposed to a 4 h treatment in serum-free media. The cells were washed and observed using a fluorescence microscope with Leica filter cube N2.1 (excitation filter BP 515–560 nm and long pass filter LP 590 nm for emission).

To determine the fluorescence intensity, the microscopic images were analyzed using a Fiji image processing package. Cells were manually segmented and specific parameters including the area and integrated fluorescence intensity were measured. The data was subsequently analyzed in a Jupyter notebook using Python, Pandas and Matplotlib packages.

For localization studies the cells were incubated with PS and subsequently loaded with 500 nM LysoSensor™ Green DND-189, LysoTracker^®^ Green DND-26, or 250 nM ER-Tracker™ Blue-White DPX for 30 min at 37 °C in the complete medium. After washing with PBS, cells were investigated in media without phenol red. Experiments were repeated three times in minimal ambient light.

For detection of ROS, 4T1 and PDT-resistant cells were sequentially incubated with temoporfin (overnight incubation) in medium and then with 3 μM CM-H_2_DCFDA probe in PBS for 30 min. After washing with PBS and adding media without phenol red, cells were exposed to a light dose of 4.4 J cm^−2^ and immediately analyzed under a fluorescence microscope. All pictures were taken under identical camera settings for each fluorescence.

### Intracellular accumulation of PS

4T1 and PDT-resistant cell lines were seeded in 96-well clear-bottom plates (BD Falcon) at 7 × 10^3^ per well and allowed to grow for 36 h. Then, the cells were treated in triplicate with KP1 (0.7 μM), KP6 (1.6 μM), or temoporfin (1 μM) overnight. The following day, the cells were washed three times with PBS and solubilized with 0.5% Triton X-100 in PBS. To determine the PS concentration, fluorescence was measured with an EnSpire plate reader (PerkinElmer) set to 423 nm excitation and 650 nm emission wavelength. Fluorescence was quantified according calibration curves of each PS. Results are expressed in fmol of PS per cell.

### Treatment with ABC transporter inhibitors

The inhibitors of ABC transporters, which were non-toxic in combination with PS, were used. Parental 4T1, KP1-, KP6-resistant clones were pre-incubated for 30 min with the inhibitors (probenecid -100 μM, MK571-100 μM, cyclosporin A -50 μM) and then KP1 or KP6 were added at a specified dose for 12–16 h. Following treatment, the cells were washed in medium without phenol red and investigated for fluorescence as described above or subjected to PDT treatment with light exposure and phototoxicity evaluation next day after irradiation.

### qPCR

Analysis of gene expression of mouse ABC transporters was performed using TaqMan assays (Applied Biosciences). cDNA was reverse-transcribed by the Maxima H minus reverse transcriptase kit (Thermo Scientific) using 1 μg total RNA as a template and oligo-dT as primers, according to manufacturer’s instructions. Assays were diluted by using 2x iTaq Universal Probes Supermix (Bio-Rad) according to the manufacturer’s instructions and 2.5 μL was used in the reaction together with 2.5 μL of diluted cDNA (concentration 4 ng/μL). Temperature profile was 95 °C for 3 min and 40 cycles of 95 °C for 5 s and 30 s at 60 °C. qPCR was performed by using Bio-Rad CFX384 Real Time PCR Instrument. The analysis of qPCR data was performed *via* the GenEx software version 6. Reference genes for normalization were identified by Normfinder; data was normalized to the *Gapdh* gene. The data was assessed for statistical analysis by using the unpaired *t*-test *via* GenEx, with the p-value < 0.05 being considered statistically significant. Ct values over 36 were considered not detected (n.d.) and were not analyzed.

### Western immunoblot analysis

Cellular extracts were prepared from parental 4T1 cells and PDT-resistant clones in RIPA buffer, separated in 6–10% sodium dodecyl sulfate-PAGE followed by electrophoretic transfer to Amersham-Hybond ECL nitrocellulose membrane. Blots were blocked for 1 h in 5% non-fat dry milk and incubated with primary antibodies. Subsequently, the blot was washed three times with washing buffer and incubated with horseradish peroxidase-conjugated donkey anti-rabbit IgG and donkey anti-mouse IgG secondary antibody (Jackson ImmunoResearch Laboratories, West Grove, PA). The membrane was then washed another five times with washing buffer and protein expression was visualized by Western lighting chemiluminescence reagent (Perkin-Elmer Life and Analytical Sciences, Wellesley, MA) or by SuperSignal West Dura reagent (Thermo Fisher Scientific, Waltham, MA). Equal protein loading and transfer was verified by Ponceau-S staining of the membrane and actin reprobing.

### RNA interference

To inhibit the ABCB1 expression in MCF-7/PacR cells, ABCB1-specific siRNA (catalog no: 4427037, ID: s10419, Life Technologies, Carlsbad, CA, USA), Opti-MEM^®^ Reduced Serum Medium (Life Technologies) and INTERFERin^®^ transfection reagent (PolyPlus-Transfection, Illkirch, FR) were used according to manufacturer’s instructions. Nonspecific siRNA (catalog no.: AM4635, Life Technologies) was used as a negative control.

The cells were seeded at a concentration of 3 × 10^5^ cells in 6 ml of antibiotics-free culture medium into a culture flask (25 cm^2^) and allowed to grow for 24 h. Afterwards, the culture medium was changed for 5 ml of fresh medium containing siRNA transfection mixture. In the transfection mixture, ABCB1 siRNA or nonspecific siRNA were diluted in Opti-MEM^®^ Reduced Serum Medium to a final concentration of 5 nM siRNA in culture medium and mixed with INTERFERin transfection reagent at 1:250 dilution. After 72-h incubation period, cells were reseeded in fresh siRNA- and taxane-free culture medium for further analysis.

### *In vivo* tumor growth

4T1 cells and cells of PDT-resistant clones (3 × 10^5^) were suspended in 0.1 mL PBS plus 0.1 mL of Matrigel (BD Biosciences, Franklin Lakes, NJ) and injected subcutaneously into depilated hind flanks of female BALB/c mice (8–12 weeks old, weight range 18–20 g). Each group consisted of four animals. The tumor growth was monitored for 32 days and tumor dimensions were determined by caliper measurements every third or fourth day. The volume of each tumor was calculated as π/6abc (where a is the longitudinal diameter, b is the short diameter and c is the thickness)^40^. All aspects of the animal experiment and husbandry were carried out in compliance with national and European regulations. The experimental protocol received approval of The Ethics Committee of the Academy of Sciences of the Czech Republic. The experiment was terminated on the 32^nd^ day to minimize animal suffering.

### Microarray analysis

The analyses were performed using 250 ng of total RNA from two separate cultures of each PDT-resistant clone and parental 4T1 cells isolated by TRIZOL Reagent (Ambion, TX) according to the manufacturer’s protocol. The quality and concentration of total RNA was measured and RNA integrity was analyzed using Agilent Bioanalyzer 2100 (Agilent, USA). Only the samples with intact RNA profiles were used for expression profiling. Microarray analysis was conducted using Affymetrix GeneChip^®^ WT PLUS Reagent Kit (WT PLUS Kit) (Affymetrix, USA) according to the standard protocol. The amount of 3.5 μg of cDNA was hybridized for 16 h at 45 °C on a Mouse Gene 2.0 ST GeneChip array (Affymetrix, USA) and scanned using the Affymetrix GeneChip Scanner 3000 7G (Affymetrix, USA). Two to four biological replicates were completed per group. It should be noted that one outlying sample was removed from the analysis. The CEL files were preprocessed using the RMA method^42^ implemented in the oligo package^43^ of the Bioconductor^44^, annotated at the gene level using the mogene20sttranscriptcluster package, and analyzed within the limma package^45^. Moderated *t*-test was used to detect transcripts differentially expressed between different strains and controls. Genes with |log2FC| > 1 and Storey’s q < 0.05^46^ were considered differentially expressed. The MIAME compliant transcription data was deposited into the ArrayExpress database (accession E-MTAB-5082)^47^. https://www.ebi.ac.uk/arrayexpress/experiments/E-MTAB-5082/.

### Statistical analysis

The data was shown as the mean value of at least three independent experiments (n) and standard deviation (±SD) represented by bars was calculated. The significance of difference was estimated by ANOVA or by *t*-test. *P,0.050, **P,0.01, ***P,0.001 represent the level of significance (P < 0.05 was considered significant). For all statistical analyses, GraphPad Software was used unless specified otherwise.

## Additional Information

**How to cite this article:** Kralova, J. *et al*. Glycol porphyrin derivatives and temoporfin elicit resistance to photodynamic therapy by different mechanisms. *Sci. Rep.* **7**, 44497; doi: 10.1038/srep44497 (2017).

**Publisher's note:** Springer Nature remains neutral with regard to jurisdictional claims in published maps and institutional affiliations.

## Supplementary Material

Supplementary Information
