# Supplementary material for: Glycol porphyrin derivatives and temoporfin elicit resistance to photodynamic therapy by different mechanisms
Source: Sci Rep. 2017 Mar 15;7:44497. doi: 10.1038/srep44497 (PMC5353759; doi:10.1038/srep44497)
Supplement: Supplementary Information [file srep44497-s1.pdf]

Supplementary Information

Glycol porphyrin derivatives and temoporfin elicit resistance to photodynamic therapy by different mechanisms

Jarmila Kralova*, Michal Kolar, Michal Kahle, Jaroslav Truksa, Sandra Lettlova, Kamila Balusikova & Petr Bartunek

*Corresponding author:

Jarmila Kralova
Institute of Molecular Genetics AS CR v.v.i.
Vídeňská 1083, 142 20 Prague 4, Czech Republic
Tel. +420-241063392
Email: kralova@img.cas.cz

Supplementary Figures S1-S7

Supplementary Tables S1-S5

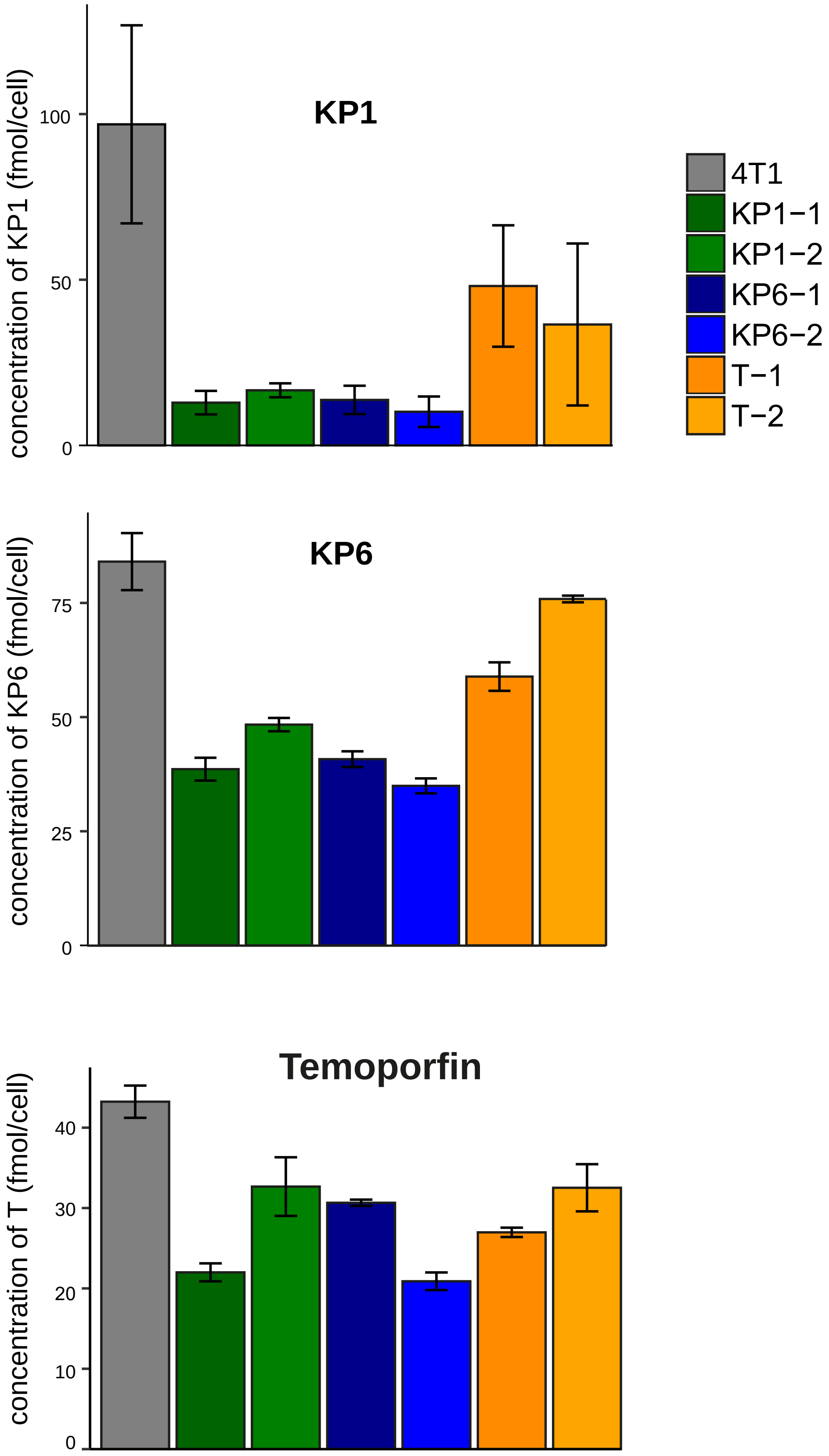

Supplementary Figure S1. Quantitative determination of intracellular accumulation of PS. 4T1 and PDT-resistant cell lines were treated with standard concentrations of PS in triplicates overnight and fluorescence of PS was determined in cell lysates with EnSpire plate reader (PerkinElmer) as described in Material and Methods.

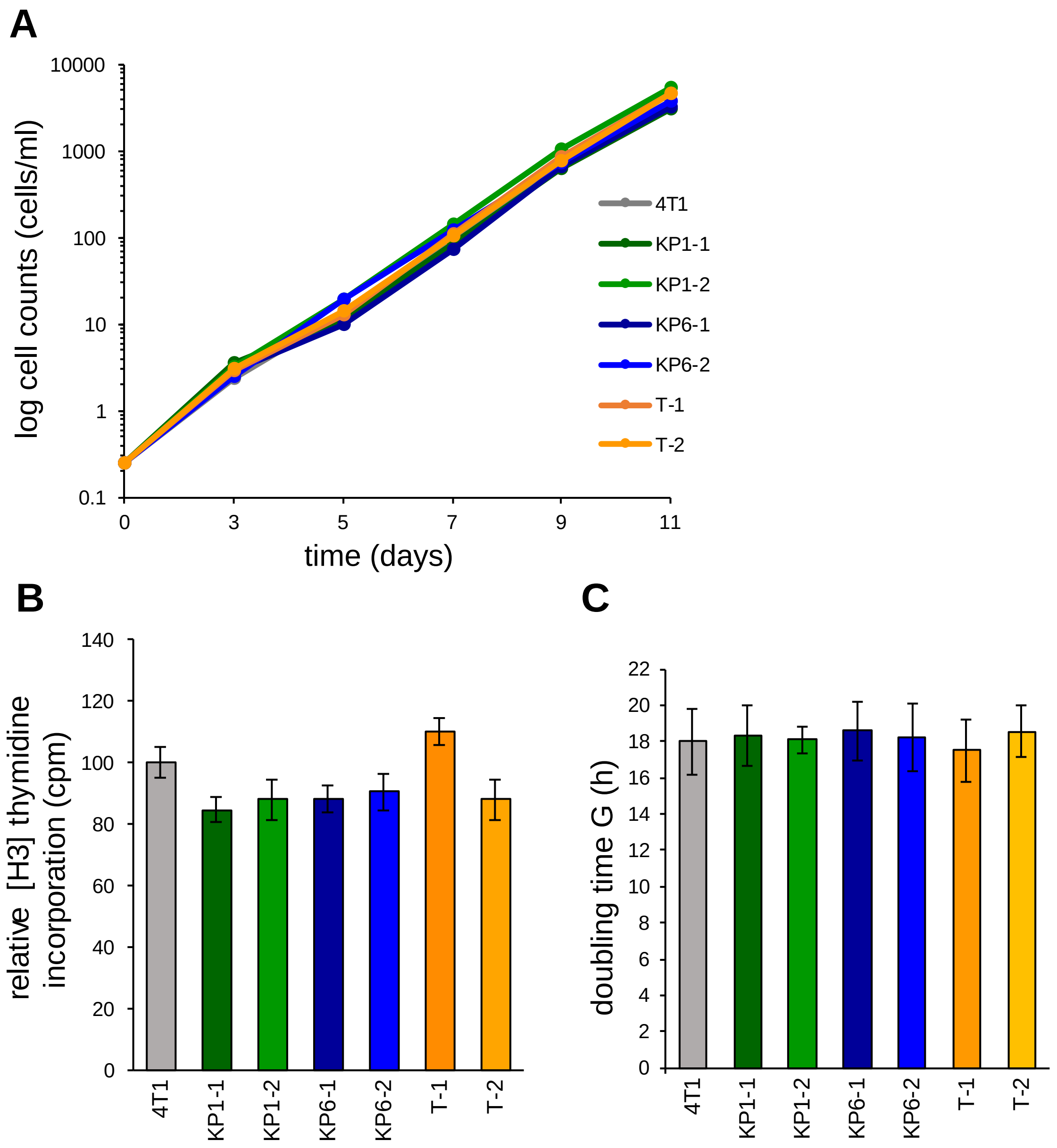

Supplementary Figure S2. Proliferative abilities of PDT-resistant clones. (**A**) Cumulative cell growth curves expressed as total cell counts in cultures of individual clones and parental cells 4T1 during the period of 11 days. (**B**) [H3] thymidine incorporation during 2 h in resistant clones relative to 4T1 cells. (**C**) Doubling time (G) as the time required for duplication of cells in the culture was calculated from data in (A) as described in Material and Methods. G represents the mean values ± standard deviation obtained from five measurements..

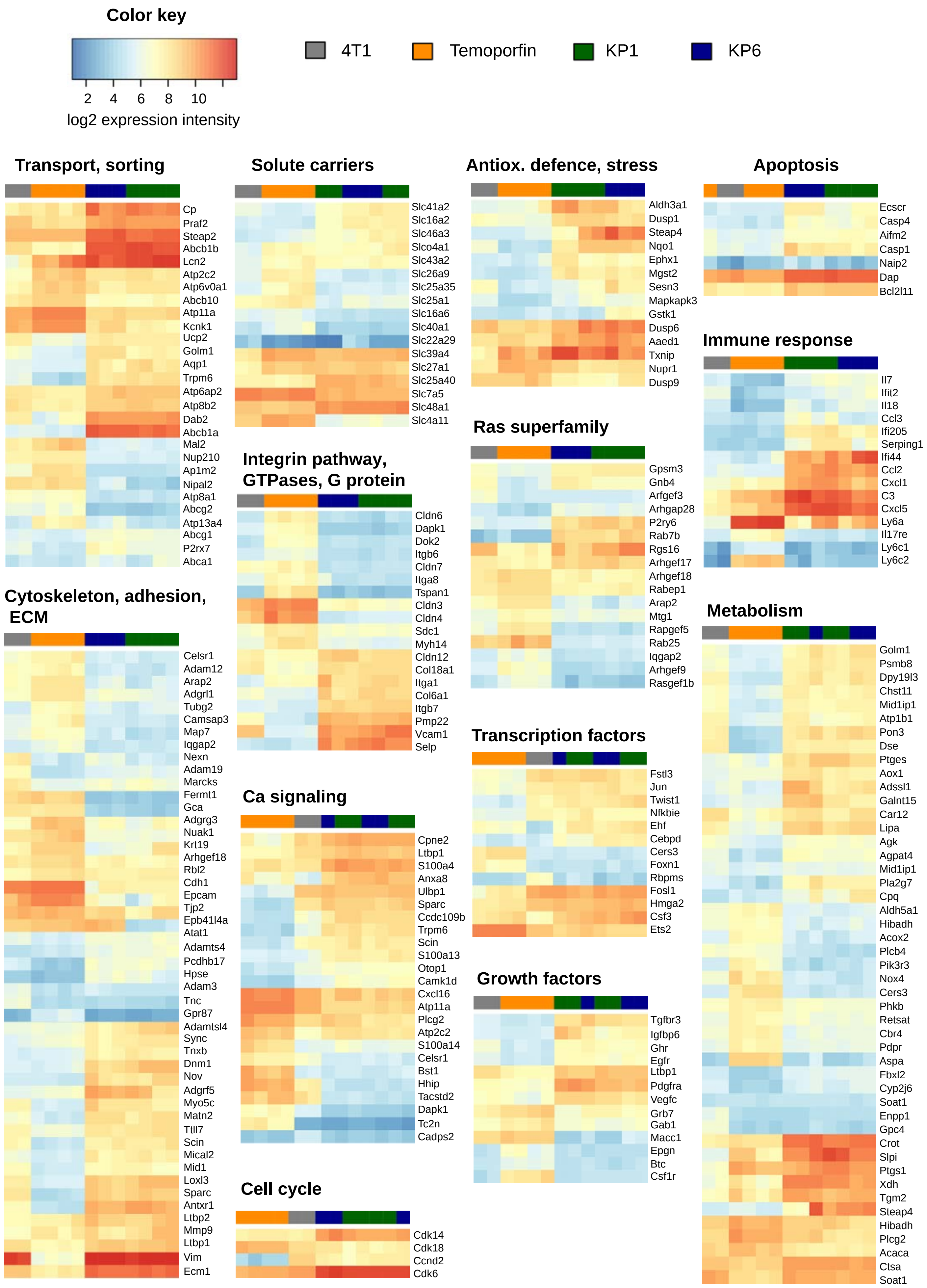

Supplementary Figure S3. Overview of thumbnail heatmaps displaying changes in the expression of the genes associated with various cellular functions. All heatmaps use the same color coding. Detailed results are given in accompanying Supplementary Table S3.

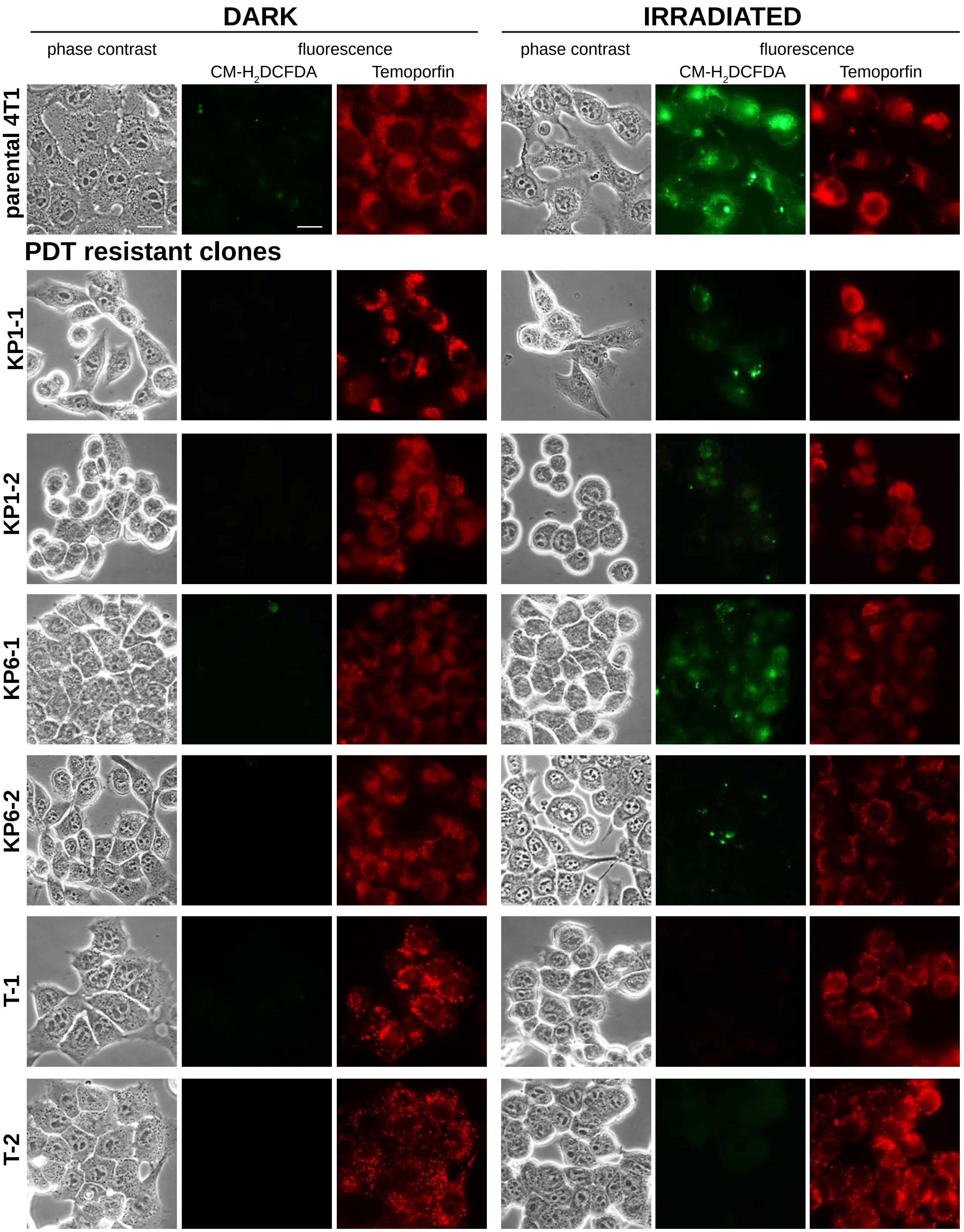

Supplementary Figure S4. Detection of ROS in PDT-resistant clones following photo-activation with temoporfin. Cells of PDT-resistant clones were incubated overnight with temoporfin (0.5 μM) and loaded with ROS-specific probe CM-H_2DCFDA (3 μM, 30 min). After washing the cells were exposed to thwe light dose 4.4 J cm^{-2}. Immediately after irradiation, CM-H_2DCFDA and temoporfin fluorescence was captured. The images of the same fields were taken under identical camera settings. Temoporfin clones did not display significant fluorescence with the ROS-detecting probe, while KP1 and KP6 were positive although less than parental 4T1 cells. Scale bars represents 10 μm.

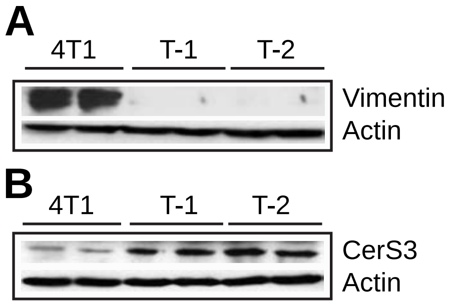

Supplementary Figure S5. **Examples of differential expression of proteins in temoporfin PDT resistant clones versus parental 4T1 cells.** The microarray analysis revealed numerous significant differences in gene expression. The corresponding deregulation on protein level is shown here for downregulation of cytoskeletal protein vimentin (A) and upregulation of ceramide synthase 3 (B). Actin served as a loading control.

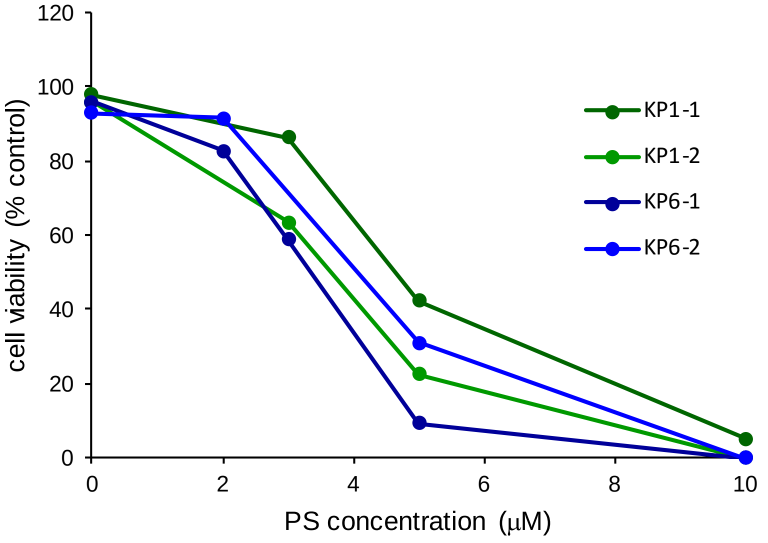

Supplementary Figure S6. Reversion of PDT resistance of KP1 and KP6 clones by increasing the PS dose. Resistant clones were treated by the corresponding KP1 and KP6 drugs in increasing concentration (2-10 µM) and exposed to the same light dose (2.5 J cm-2). Cell viability was evaluated after 24 h as described in Material and Methods. Cells treated with PS but without light exposure were used as controls.

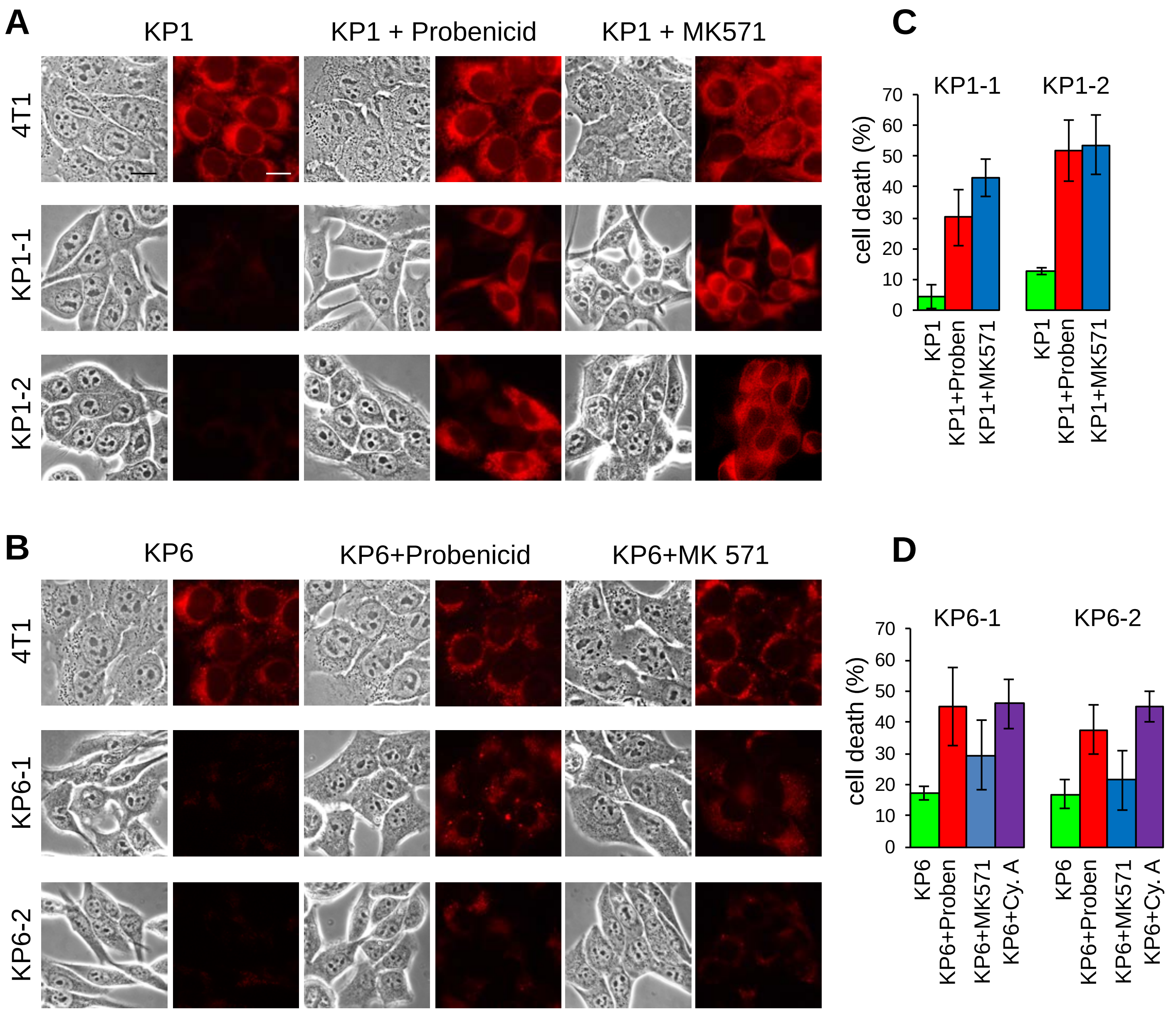

Supplementary Figure S7. The inhibitors of ABC transporters partly increased porphyrin fluorescence and phototoxicity in KP1 and KP6 PDT resistant clones. The parental 4T1 and KP1 and KP6 resistant cells were exposed to the treatment of either KP1 and KP6 alone or were pre-incubated for 30 min with inhibitors of efflux transporters (probenicid -100 µM, MK571-100 µM, and cyclosporin A - 50 µM) and then incubated with porphyrins for additional 16 h. After washing the cells were subjected to fluorescence microscopy (**A, B**) or photodynamic treatment with light to induce phototoxicity (**C, D**). The phototoxicity evaluated 24h after PDT treatment is expressed as the percentage of dead cells. The combination of cyclosporin A with KP1 was toxic, therefore was not included in C. The graphs represent the mean of at least three experiments with standard deviation. Scale bar represents 10 µm. The inhibitors restored PS fluorescence in some cells and increased sensitivity to PDT treatment in PDT resistant clones but did not affect parental cells.

Supplementary Tables

Supplementary Table S1. Treatment conditions for isolation of resistant cells by repeated PDT cycles.

PS	Concentr.	Incubation time (h)	Wavelength λ (nm)	Power density (mW cm^{-2})	Light dose (J cm^{-2})	No. of cycles	Clones	Resistance index
KP1	0.7 μM	16	500-520	0.7	2-13	10	KP1-1<br>KP1-2	20-27
KP6	1.6 μM	16	500-520	0.7	0.5-7	15	KP6-1<br>KP6-2	20-23
Temoporfin	1 μM	16	620-660	3.7	7.5-26	20	T-1<br>T-2	3.5-4

Supplementary Table S2. Effective light doses, LD_{50} and resistance indexes of PDT-resistant clones relative to parental 4T1 cells. The resistance indexes (RI) are defined as LD_{50} of resistant clone/LD_{50} of parental cells. Each value is a mean of at least four experiments.

Cells	LD_{50} (J cm^{-2})				Resistance index (RI)			
	KP1	KP6	Temoporfin	Photofrin	KP1	KP6	Temoporfin	Photofrin
4T1	0.45±0.03	0.29±0.09	7.5±0.67	3.88±1.4	1.0	1.0	1.0	1.0
KP1-1	12.2±0.72	2.54±0.31	16.4±0.45	1.63±0.71	27.1	8.6	2.2	0.42
KP1-2	9.07±0.25	0.45±0.12	7.57±0.6	1.64±0.29	20.1	1.6	1.0	0.42
KP6-1	>13	6.79±0.55	15.8±0.31	2.92±0.05	>28	23.4	2.1	0.75
KP6-2	>10	5.88±0.79	12.8±0.79	2.48±0.37	>22	20.3	1.7	0.64
T-1	0.49±0.19	0.48±0.05	31.0±2.3	4.4±0.95	1.08	1.65	4.1	1.13
T-2	0.43±0.11	0.52±0.05	25.9±3.45	3.46±1.43	0.95	1.8	3.45	0.89

Supplementary Table S3. Expression intensities of genes clustered to functional classes. Differentially expressed genes in KP1, KP6 and temoporfin PDT-resistant cells versus parental 4T1 cells. The classification is arbitrary and some subclasses or individual genes could be classified differently.

TRANSPORTERS

		log2 fold change		
		KP1	KP6	Temoporfin
**ATP-binding cassette transporters**				
Abcb1a	ATP binding cassette subfamily B, member 1	5.52	5.8	
Abcb1b	ATP binding cassette subfamily B, member 1	3.45	3.25	-1.24
Abca1	ATP binding cassette subfamily A, member 1	1.56	1.34	
Abcg1	ATP binding cassette subfamily G, member 1	0.5	1.06	
Abcg2	ATP binding cassette subfamily G, member 2	-2.26	-2.48	
Abcb10	ATP binding cassette subfamily B, member 10	-0.5	-0.5	0.5
**Ion transporters, channels**				
Lcn2	Lipocalin; Iron-trafficking protein	5.46	4.99	3.56
Atp2c2	ATPase, Ca++ transporting, type 2C, member 2	1.27	1.22	2.24
Atp6v0a1	ATPase, H+ transporting, lysosomal V0 subunit A1	0.5	0.5	1.5
Trpm6	transient receptor potential cation channel, subfamily M member	2.36	2.16	-1.84
Cp	ceruloplasmin; copper ion binding and ferroxidase activity, iron transport	3.01	3.04	
Steap2	six transmembrane epithelial antigen of prostate 2; transport of sugars, bile salts and organic acids, metal ions and amine compounds, oxidoreductase activity	1.7	2	
P2rx7	purinergic receptor P2X, ligand-gated ion channel	0.5	1.32	-0.5
Atp6ap2	ATPase, H+ transporting, lysosomal accessory protein 2, acidification of intracellular compartments, and cellular pH homeostasis	0.5	0.5	-0.5
Kcnk1	potassium channel, subfamily K, member 1	-2.27	-1.26	0.5
Atp11a	ATPase, class VI, type 11A; transport calcium across membranes	-1.26	-1.16	0.5
Atp13a4	ATPase type 13A4			2.43
Atp1b1	ATPase, Na+/K+ transporting, beta 1 polypeptide			-2.03
Ucp2	uncoupling protein 2; mitochondrial proton carrier			-1.52
Nipal2	NIPA-like domain containing 2; magnesium ion transmembrane transporter activity	-4.57	-3.19	
**Macromolecule transporters**				
Dab2	disabled; mitogen-responsive phosphoprotein adapter protein that functions as clathrin-associated sorting protein	2.55	2.51	-0.5
Golm1	Golgi membrane protein 1; sorting and modification of proteins exported from ER	0.5	1.75	-1.32
Praf2	PRA1 domain family 2; ER/Golgi transport and vesicular traffic	1.1	1.1	-0.5
Atp8b2	ATPase, class I, type 8B, member 2; phospholipid translocation	0.5	0.5	-0.5
Ap1m2	adaptor protein complex AP-1, mu 2 subunit; clathrin-associated adaptorprotein, protein sorting in the trans-Golgi network and endosomes	-1.82	-1.9	1.93
Mal2	T cell differentiation protein 2; required for transcytosis	-2.09	-2.24	1.41
Atp8a1	ATPase, aminophospholipid transporter (APLT), class I, type 8A, member 1	-1.92	-1.98	
Nup210	nucleoporin 210; flow of macromolecules between the nucleus and the cytoplasm	-2.11	-1.9	
**Solute carriers**				
Slco4a1	solute carrier organic transporter family 4, member 1	1.68	1.33	1.53
Slc27a1	solute carrier family 27, member 1	1.47	1.73	1.72
Slc31a2	solute carrier family 31, member 2	1.14	1.5	1.47
Slc39a4	solute carrier family 39, member 4	1.35	1.44	1.94
Slc43a2	solute carrier family 43, member 2	1.23	1.17	1.36
Slc46a3	solute carrier family 46, member 3	2.18	2.14	0.5
Slc41a2	solute carrier family 41, member 2	1.25	1.73	-0.5
Slc12a6	solute carrier family 12, member 6	1.77	1.43	
Slc16a2	solute carrier family 16, member 2	1.94	2.33	
Slc25a40	solute carrier family 25, member 40	2.16	2.36	
Slc48a1	solute carrier family 48, member 1	1.29	1.37	
Slc4a11	solute carrier family 4, sodium bicarbonate transporter-like, member 11	-2.75	-2.15	1.15
Slc7a5	solute carrier family 7, member 5	-1.03	-1.06	
Slc16a6	solute carrier family 16, member 6	-1.1	-1.2	
Slc25a15	solute carrier family 25, member 15	-1.47	-1.2	0.5
Slc40a1	solute carrier family 40, member 1	-0.5	-0.5	0.5
Slc25a35	solute carrier family 25, member 35			1.95
Slc26a9	solute carrier family 26, member 9			1.9
Slc22a29	solute carrier family 22, member 29	-1.85		-1.77
Slc29a2	solute carrier family 29, member 2	-1.08	-1.07	-0.5

CELLULAR DEFENSE

		KP1	KP6	Temoporfin
**Antioxidant defense, detoxification pathways**				
Aldh3a1	aldehyde dehydrogenase family 3, subfamily A1	3.95	2.78	0.5
Txnip	thioredoxin interact. prot	4.05	3.33	2.4
Aaed1	AhpC/TSA antioxidant enzyme domain containing 1	1.77	2.13	
Mgst2	microsomal glutathione S-transferase 2	2.86	2.88	
Steap4	STEAP family member 4; oxidoreductase activity	3.4	6	
Ephx1	epoxide hydrolase 1; microsomal biotransformation enzyme that converts epoxides from the degradation of aromatic compounds to trans-dihydrodiols which can be conjugated and excreted from the body	2.36	1.96	
Gstk1	glutathione S-transferase kappa 1		2.35	
Nqo1	NAD(P)H dehydrogenase, quinone 1	2	2.41	-2
**Stress response**				
Dusp1	dual specificity phosphatase 1; response to environmental stress	1.69	1.37	
Dusp6	dual specificity phosphatase 6; inactivates ERK2	0.5	1.68	-0.5
Dusp9	dual specificity phosphatase 9; specificity for ERK family	-1.61	-0.5	
Sesn3	sestrin 3; stress-induced protein, reduces the levels of intracellular reactive oxygen species	-0.5		-2.16
GRP87	G protein-coupled receptor 87; important for p53-dependent cell survival in response to genotoxic stress			2.65
Mapkapk3	mitogen-activated protein kinase-activated protein kinase 3			-1.5
Nupr1	nuclear protein transcription regulator 1; Chromatin-binding protein that converts stress signals into a program of gene expression that empowers cells with resistance to the stress	0.5	0.5	1.82
**Arachidonic acid and prostaglandin metabolism**				
Ptgs1	prostaglandin-endoperoxide synthase 1; prostaglandin G/H synthase and cyclooxygenase	2.9	3.55	3.59
Ptges	prostaglandin E synthase	2.73	2.65	
Plcg2	phospholipase C, gamma 2			1.58
Pik3r3	phosphatidylinositol 3 kinase, regulatory subunit, polypeptide 3 (p55)			1.96
Cyp2j6	cytochrome P450, family 2, subfamily j, polypeptide 6			-1.55
**DNA repair**				
Trex2	three prime repair exonuclease 2; double-stranded DNA break repair			2.15
Chek1	checkpoint kinase 1; checkpoint-mediated cell cycle arrest and activation of DNA repair	-1.68	-1.26	
**Apoptosis**				
Casp1	caspase 1	1.68	2.45	
Casp4	caspase 4, apoptosis-related cysteine peptidase	1.67	1.9	
Bcl2l11	BCL2-like 11; apoptosis facilitator	1.66	1.49	

Ecscr	endothelial cell surface expressed chemotaxis and apoptosis regulator	1.38	2.39	
Naip2	NLR family, apoptosis inhibitory protein 2	0.5	2.14	1.25
Aifm2	apoptosis-inducing factor, mitochondrion-associated 2	1.34	1.26	?
Dap	death-associated protein; positive regulator apoptosis, negative autophagy	1.26	1.22	-0.5
**Immune response**				
Ly6c1	lymphocyte antigen 6 complex, locus C1			2.52
Ly6c2	lymphocyte antigen 6 complex, locus C2			6.2
Ly6a	lymphocyte antigen 6 complex, locus A	2.5	2.75	6.07
C3	complement component 3	4.42	3.5	2.16
Il7	interleukin 7	0.5	0.5	-1.89
Il17re	interleukin 17 receptor E			1.89
Il18	interleukin 18			-1.81
Ifit2	interferon-induced protein with tetratricopeptide repeats 2 ; Interferon signaling, can promote apoptosis			-2.05
Ifi44	interferon-induced protein 44; microtubule associated protein	4.44	5.62	
Ifi205	interferon activated gene 205	3.94	3.2	
Cxcl1	chemokine (C-X-C motif) ligand 1	3.92	2.99	0.5
Cxcl3	chemokine (C-X-C motif) ligand 3	4.96	2.58	
Cxcl5	chemokine (C-X-C motif) ligand 5	4.37	3.96	0.5
Cxcl16	chemokine (C-X-C motif) ligand 16; calcium mobilization, signaling	-0.5	-0.5	0.5
Ccl2	chemokine (C-C motif) ligand 2	4.44	4.38	
Ccl3	chemokine (C-C motif) ligand 3	1.87		
Serping1	serine (or cysteine) peptidase inhibitor, clade G, member 1; regulation of complement cascade	3.29	3.17	
**CYTOSKELETON, ADHESION, ECM**				
**Cytoskeleton, extracellular matrix**				
Sync	Syncoilin; Intermediate Filament Protein	2.45	2.01	1.1
Dnm1	dynamin; microtubule-associated force-producing protein, vesicular trafficking processes	3.85	3.05	
Antxr1	anthrax toxin receptor 1; tumor-specific endothelial marker, docking protein, transmembrane signaling receptor activity and collagen binding, Interacts with extracellular matrix proteins and with the actin cytoskeleton	2.89	2.51	-2,37
Mical2	microtubule associated monooxygenase, calponin and LIM domain containing 2; actin binding and oxidoreductase aktivity	1.46	1.25	-1.32
Atat1	alpha tubulin acetyltransferase 1	0.5	0.5	-0.5
Tubg2	Tubulin g2			2
Map7	microtubule-associated protein 7	-1.63	-0.5	0.5
Ttll7	tubulin tyrosine ligase-like family member 7			-1.1
Marcks	myristoylated alanine rich protein kinase C substrate; filamentous (F) actin cross-linking protein	-1.3	-1.49	-2.13
Arhgef18	rho/rac guanine nucleotide exchange factor (GEF) 18; cytoskeletal rearrangements	-0.5	-0.5	0.5
Epb41l4a	erythrocyte membrane protein band 4.1 like 4a; interaction between the cytoskeleton and plasma membrane	-4.15		
Vim	vimentin			-6.09
Scin	scinderin SCIN; regulation of actin cytoskeleton			-2.48
Mid1	midline 1; transcription regulator, associating with microtubules			-2
Myo5c	myosin VC; actin binding and motor activity		0.5	-2.13
Iqgap2	IQ motif containing GTPase activating protein 2; actin binding			1.2
Krt19	keratin19; structural constituent of cytoskeleton		-0.5	1.44
Rbl2	Retinoblastoma-like 2; potent inhibitor of E2F-mediated trans-activation, tumor supressor	0.5	0.5	1.14
PAK1	P21 Prot. (Cdc42/Rac)-Activated Kinase 1			-1.37
**Cell adhesion, cell junctions**				
Steap1	six transmembrane epithelial antigen of the prostate 1; surface antigen expressed at cell-cell junctions	2.2	2.35	
Adgrf5	adhesion G protein-coupled receptor F5	2.93	3.99	0.5
Vcam	vascular cell adhesion mol. 1	1.58	0.5	-3.61
Gca	granalcin; role in focal adhesions	-4.39	-3.97	
Cdh1	cadherin 1	-3.31	-3.75	
Epcam	epithelial cell adhesion molecule	-3.32	-2.6	
Nexn	nexilin; filamentous actin-binding protein that may function in cell adhesion and migration	-2.95	-1.82	-2.94
Nuak1	NUAK family, SNF1-like kinase, 1; involved in cell adhesion	-1.8	-1.68	0.5
Camsap3	calmodulin regulated spectrin-associated protein family member	-0.5	-0.5	0.5
Arap2	ArfGAP with RhoGAP domain, ankyrin repeat and PH domain 2; regulate focal adhesion dynamics	-1.9	-1.35	0.5
Celsr1	cadherin, EGF LAG seven-pass G-type receptor 1 (flamingo homolog, Drosophila); receptor for contact mediated communication	-1.47	-1.36	0.5
Gpr87	G protein-coupled receptor 87			2.1
Adgrg3	adhesion G protein-coupled receptor G3			2.3
Adgrl1	adhesion G protein-coupled receptor L1			1.36
Pcdhb17	protocadherin beta 17			-1.66
**Extracellular matrix**				
Mmp9	matrix metallopeptidase 9	2.05	0.5	0.5
Ltbp2	latent transforming growth factor beta binding protein 2; extracellular matrix protein	1.83	2.05	0.5
Loxl3	lysyl oxidase-like 3; degradation of ECM	1.3	1.25	-2.5
Ecm1	extracellular matrix protein 1	2.37	2.22	-1.2
Ltbp1	latent transforming growth factor beta binding protein 1; sequestering of TGFbeta in ECM	0.5	0.5	-1.25
Matn2	matrilin 2; involved in the formation of filamentous networks in the extracellular matrices	1.44	1.79	-1.55
Tnxb	tenascin XB; extracellular matrix glycoprotein, anti-adhesive effects	2.53	2.43	
Hpse	heparanase; enzyme that cleaves heparan sulfate proteoglycans to permit cell movement through remodeling of the extracellular matrix	1.9	2.6	
Nov	nephroblastoma overexpressed gene; associate with the extracellular matrix	3.4	3.4	
Adamts4	a disintegrin-like and metallopeptidase (reprolysin type) with thrombospondin type 1 motif	1.93	1.68	
Adamtsl4	ADAMTS-like 4, disintegrin and metalloproteinase with thrombospondin motifs-like gene family	2.54	1.34	
Fermt1	fermitin family homolog 1; integrin signaling and linkage of the actin cytoskeleton to the ECM	-5	-5.11	
Adam12	a disintegrin and metallopeptidase domain 12 (meltrin alpha)	-2.28	-2	
Adam19	disintegrin and metallopeptidase domain 19 (meltrin beta)	-1.25	-1.53	-2.53
Adam3	a disintegrin and metallopeptidase domain 3 cyritestin, membrane anchored cell surface adhesion protein, metalloproteinase with thrombospondin motifs-like gene family	-1.16	-0.5	-1.51
Tnc	tenascin C; extracellular matrix protein	-2.12	-1.9	-1.99
Tjp2	tight junction protein 2	-1.63	-0.5	
Sparc	secreted acidic cysteine rich glycoprotein; involved in extracellular matrix remodeling			-3.76
**Integrin pathway**				
Pmp22	peripheral myelin protein 22	2.31	2.47	-2.21
Selp	selectin platelet; Ca(2+)-dependent receptor, integrin signaling	5.88	5.47	
Col6a1	collagen, type VI, alpha 1	2.03	1.77	-0.5
Vcam1	vascular cell adhesion molecule 1; integrin binding and primary amine oxidase activity	1.6	1.49	-3.61
Itga1	integrin alpha 1	3.44	3.58	0.5
Itgb3	integrin beta 3	1.92	2.12	0.5

Itgb7	integrin beta 7	3.28	3.22	
Cldn12	claudin 12	0.5	1.38	-0.5
Itga8	integrin alpha 8	-1.3	-1.2	1.38
Dapk1	death associated protein kinase 1	-1.55	-1.48	1.74
Cldn3	claudin 3	-2.75	-2.36	1.2
Cldn4	claudin 4	-3.8	-4.22	0.5
Cldn7	claudin 7	-2.32	-2.41	
Cldn6	claudin 6			3.19
Itgb6	integrin beta 6			1.1
Myh14	myosin, heavy polypeptide 14; calmodulin binding and microfilament motor activity			2.42
Col18a1	collagen, type XVIII, alpha 1			-1.25
Sdc1	syndecan 1			1.3
Tspan1	tetraspanin 1; positive regulation of endocytosis			4.73
Dok2	docking protein 2			2.27
PAK1	P21 Prot. (Cdc42/Rac)-Activated Kinase 1			-1.37

CELL GROWTH

Ras superfamily, GTPase, G-protein signaling

Rab7b	member RAS oncogene family, controls vesicular trafficking from endosomes to the trans-Golgi network	5.01	5.03	
P2ry6	pyrimidinergic receptor P2Y, G-protein coupled, 6; G-protein coupled receptor activity and UDP-activated nucleotide receptor activity	3.5	2.99	
Rab25	member RAS oncogene family, regulation of cell survival, epithelial morphogenesis, membrane trafficking	-3.46	-3.53	
Rgs16	regulator of G-protein signaling 16		-2.16	-2.16
Mtg1	mitochondrial GTPase activity			1.22
Arap2	ArfGAP with RhoGAP domain, ankyrin repeat and PH domain 2	-1.9	-1.35	1.14
Rasgef1b	RasGEF domain family, memb. 1B	-1.3	-0.5	1.45
Rapgef5	Rap guanine nucleotide exchange factor (GEF) 5	-1.55	-1.76	1.62
Rabep1	rabaptin, RAB GTPase binding effector protein 1			1
Arhgef9	CDC42 guanine nucleotide exchange factor (GEF) 9			1.44
Arhgef18	rho/rac guanine nucleotide exchange factor (GEF) 18	-0.5	-0.5	0.5
Arhgap28	Rho GTPase activating protein 28	-0.5	-0.5	0.5
Arhgap29	Rho GTPase activating protein 29	-3.37	-2.41	-3.94
Arhgef17	Rho guanine nucleotide exchange factor (GEF) 17	0.5	0.5	-0.5
Gpsm3	G-protein signalling modulator 3	0.5	0.5	-0.5
Iqgap2	IQ motif containing GTPase activating protein 2			1
Gnb4	guanine nucleotide binding protein (G protein), beta 4; signal transducer activity			-2.07
Arfgef3	ARFGEF family member 3; positive regulation of GTPase activity	-1.49	-1.61	-1.57

Metabolism

Slpi	secretory leukocyte peptidase inhibitor; inhibitor which protects epithelial tissues from serine protease	5.42	5.26	0.5
Xdh	xanthine dehydrogenase	4.31	3.98	0.5
Tgm2	transglutaminase 2, C polypeptide; Catalyzes the cross-linking of proteins and the conjugation of polyamines to proteins	2.74	2.66	0.5
Aox1	aldehyde oxidase 1	1.97	1.98	0.5
Cpq	carboxypeptidase Q; metallodipeptidase aktivity	1.73	3.5	0.5
Galnt15	UDP-N-acetyl-alpha-D-galactosamine:polypeptide N-acetylgalactosaminyltransferase 15	3.43	2.98	
Crot	carnitine O-octanoyltransferase; lipid metabolism and fatty acid beta- oxidation	3.28	3.56	
Adssl1	adenylosuccinate synthetase like 1	2.9	2.77	
Steap4	metalloreductase and oxidoreductase activity, maybe involved in adipocyte development and metabolism	3.41	6	
Aspa	aspartoacylase			4.52
Acox2	acyl-Coenzyme A oxidase 2			2
Cers3	ceramide synthase 3; Sphingolipid metabolism			3.41
Cbr4	carbonyl reductase 4; NAD(P)H dehydrogenase (quinone) activity			1.27
Retsat	retinol saturase (all-trans retinol 13,14 reductase); oxidoreductase activity, metabolism of vitamin A			1.37
Pon3	paraoxonase 3			-3.31
Dse	dermatan sulfate epimerase			-3.83
Agk	acylglycerol kinase			-1.22
Atp1b1	ATPase, Na+/K+ transporting, beta 1 polypeptide			-2.03
Agpat4	1-acylglycerol-3-phosphate O-acyltransferase 4; lysophosphatidic acid acyltransferase, delta			-1.72
Lipa	lysosomal acid lipase A	1.1	1.38	-1.38
Pla2g7	phospholipase A2, group VII; platelet-activating factor acetylhydrolase, plasma	1.93	2.41	-0.5
Chst11	carbohydrate sulfotransferase 11; Chondroitin sulfate/dermatan sulfate metabolism	0.5	0.5	-1.4
Golm1	Golgi Membrane Protein 1; lipid metabolism	0.5	1.75	-1.32
Fbxl2	F-box and leucine-rich repeat protein 2; ubiquitin-protein transferase activity and calmodulin binding	1.47	1.67	-1.21
Ctsa	cathepsin A; protein recycling within the lysosome	0.5	0.5	-0.5
Plcg2	phospholipase C, gamma 2			1.54
Soat1	sterol O-acyltransferase 1			-1.61
Plcb4	phospholipase C, beta 4	-1.94	-2.07	-
Nox4	NADPH oxidase 4	-0.5	-0.5	0.5
Acaca	acetyl-Coenzyme A carboxylase alpha	-0.5	-0.5	0.5
Aldh5a1	aldhehyde dehydrogenase family 5, subfamily A1	-0.5	-0.5	0.5
Phkb	phosphorylase kinase beta; glycogen catabolism, calmodulin binding and hydrolase activity			1.14
Retsat	retinol saturase; all-trans retinol 13,14 reductase			1.37
Dpy19l3	dpy-19-like 3; transferring glycosyl groups		0.5	-2.57
Hibadh	3-hydroxyisobutyrate dehydrogenase; NAD binding and 3-hydroxy-isobutyrate dehydrogenase activity		-0.5	1.02
Pdpr	pyruvate dehydrogenase phosphatase regulatory subunit; oxidoreductase activity and aminomethyltransferase activity			1.45
Enpp1	ectonucleotide pyrophosphatase/phosphodiesterase	-1.82	-1.74	-1.92
Gpc4	glypican 4; Cell surface heparan sulfate proteoglycans	-2.28	-2.18	-2.53
Agk	acylglycerol kinase; Glycerolipid metabolism; NAD+ kinase activity and ceramide kinase activity			-1.05
Mid1ip1	Mid1 interacting protein 1 (gastrulation specific G12-like (zebrafish)); regulation of lipogenesis			-1.92
Psmb8	proteasome (prosome, macropain) subunit, beta type 8; large multifunctional peptidase 7		1.51	-1.36
Car12	carbonic anyhydrase 12; catalyze the reversible hydration of carbon dioxide to neutralize the abnormally acidic pH of the extracellular environment induced during hypoxia	?		-1.56

NO

Nos2	nitric oxide synthase 2, inducible	4.59	3.02	2.26
Ddah2	dimethylarginine dimethylaminohydrolase 2; NOS inhibitor	0.5	0.5	-0.5
Gch1	GTP cyclohydrolase 1	2.29	2.63	0.5

Transcription factors, transcription regulation

Ehf	ets homologous factor; transcriptional repressor and may be involved in epithelial differentiation and carcinogenesis	3.91	3.13	2.47
Csf3	colony stimulating factor 3; controls the production, differentiation, and function of granulocytes, growth factor activity	2.73	2.35	0.5
Cebpd	CCAAT/enhancer binding protein delta; bZIP transcription factor involved in immune and inflammatory responses	2.1	2.6	
Cers3	ceramide synthase 3; transcription factor activity and sphingosine N-acyltransferase activity			3.06

Ets2	E26 avian leukemia oncogene 2, 3' domain; Ras and ERK signaling			2
Foxn1	forkhead box N1; transcription regulator			2.66
Twist1	twist basic helix-loop-helix transcription factor 1			-1.94
Hmga2	high mobility group AT-hook 2; transcriptional regulating factor			-1.2
Jun	jun proto-oncogene			-1.2
Fosl1	fos-like antigen 1			-2.37
Nfkbie	nuclear factor of kappa light polypeptide gene enhancer in B cells; inhibits NF-kappa-B by complexing with and trapping it in the cytoplasm			-1.51
Fstl3	follistatin-like 3; antagonizing protein for members of the TGF-beta family			-2.04
Rbpms	RNA binding protein gene with multiple splicing; Acts as a coactivator of transcriptional activity. Required to increase TGFB1/Smad-mediated transactivation	-2.3	-2.94	-2.68
**Growth factors, receptors, associated proteins**				
Pdgfra	platelet derived growth factor receptor, alpha polypeptide; catalytic receptors that have intracellular tyrosine kinase activity	2.75	2.5	
Igfbp6	insulin-like growth factor binding protein 6; prolongs the half-life of the IGFs and have been shown to either inhibit or stimulate the growth promoting effects of the IGFs	2.54	2	
Tgfbr3	transforming growth factor, beta receptor III; binds to TGF-beta, capturing and retaining TGF-beta for presentation to the signaling receptors	2.82	2.9	
Vegfc	vascular endothelial growth factor C; growth factor activity and vascular endothelial growth factor receptor 3 binding	1.7	1.5	
Egfr	epidermal growth factor receptor	1.16	1.3	-0.5
Ltbp1	latent transforming growth factor beta binding protein 1	0.5	0.5	-1.25
Btc	betacellulin, epidermal growth factor family member			1.32
Epgn	epithelial mitogen; ligands for the epidermal growth factor receptor and play a role in cell survival, proliferation and migration			1.6
Grb7	growth factor receptor bound protein 7			1.08
Gab1	growth factor receptor bound protein 2-associated protein 1			1.1
Ghr	growth hormone receptor; cytokine receptor activity, metastasis associated in colon cancer 1, growth factor activity			-1.5
Macc1	metastasis associated in colon cancer 1; key regulator of the hepatocyte growth factor	-4.75	-3.53	
Ctgf	connective tissue growth factor; growth factor activity and integrin binding	-2.62	-2.01	-0.5
Csf1r	colony stimulating factor 1 receptor			3.51
**Ca2+ binding, transport, homeostasis, signaling**				
Anxa8	annexin A8; calcium ion binding and calcium-dependent phospholipid binding	3.78	3.71	0.5
Atp2c2	ATPase, Ca++ transporting, type 2C, member 2	1.27	1.22	2.24
Trpm6	transient receptor potential cation channel, subfamily M, member	2.36	2.16	-1.84
Ccdc109b	coiled-coil domain containing 109B; Ca transport, homeostais, signaling	1.89	1.59	-2.05
Camk1d	calcium/calmodulin-dependent protein kinase ID; Ca signaling	1.62	1	-1.41
Ltbp1	latent transforming growth factor beta binding protein 1; calcium ion binding, signaling	0.5	0.5	-1.25
Cpne2	copine II; calcium-dependent membrane-binding proteins	0.5	0.5	-1.88
S100a4	S100 calcium binding protein A4	2.13	1.91	
S100a13	S100 calcium binding protein A13	2.07	1.7	
Tacstd2	tumor-associated calcium signal transducer 2; receptor transducing Ca signals	-3.36	-3.5	
Hhip	Hedgehog-interacting protein	-3.01	-2.82	2.17
Dapk1	death associated protein kinase 1; calcium/calmodulin-dependent serine/threonine kinase, Ca signaling	-1.55	-1.48	1.74
Celsr1	cadherin, EGF LAG seven-pass G-type receptor 1 (flamingo homolog, Drosophila); Ca signaling	-1.47	-1.36	1
Atp11a	ATPase, class VI, type 11A; transport calcium across membranes	-1.26	-1.16	0.5
Cxcl16	chemokine (C-X-C motif) ligand 16; calcium mobilization, signaling	-0.5	-0.5	0.5
Cadps2	Ca2+-dependent activator protein for secretion 2	-1.43	-1.36	-1.48
Tc2n	tandem C2 domains, nuclear; Ca binding			4.89
Bst1	bone marrow stromal cell antigen 1; calcium release from intracellular stores, Ca signaling			4.32
S100a14	S100 calcium binding protein A14			2.46
Plcg2	phospholipase C, gamma; using calcium as a cofactor, Ca signaling			1.53
Sparc	secreted acidic cysteine rich glycoprotein; Ca binding, homeostasis	-3.76		
Ulbp1	UL16 binding protein 1; mitochondrial inner membrane calcium uniporter; Ca binding	-3.68		
Scin	scinderin SCIN; Ca(2+)-dependent actin-severing, Ca binding, signaling			-2.48
Otop1	otopetrin 1; calcium homeostasis			-1.47
**Cell cycle, proliferation**				
Cdk6	cyclin-dependent kinase 6	1.95	2.16	
Cdk14	cyclin-dependent kinase 14	2.3	2.87	
Cdk18	cyclin-dependent kinase 18	-1.12	-0.5	1.05
Ccnd2	cyclin D2	-1.34	-0.5	-4.76
**Hypoxia**				
Egln1	egl-9 family hypoxia-inducible factor 1; Regulation of Hypoxia-inducible Factor (HIF) by Oxygen			0.5
Egln3	egl-9 family hypoxia-inducible factor 3; Regulation of Hypoxia-inducible Factor (HIF) by Oxygen	1.64	2.04	0.5
**Others**				
Aqp1	aquaporin; water channel protein	2.46	2.02	
Tnip3	TNFAIP3 interacting protein 3; inhibits NF-kappa-B activation	4.08	4.09	
Lrp1	low density lipoprotein receptor-related protein 1; (endocytic receptor	1.53	1.66	
Acap1	ArfGAP with coiled-coil, ankyrin repeat and PH domains 1; endocytosis	1.22	1.61	2.1
Deptor	DEP domain containing MTOR-interacting protein; negative regulator of the mTORC1 and mTORC2 signaling pathways, Akt signaling	1.88	2.22	1.36
Scara5	scavenger receptor class A, member 5; putative ferritin receptor that mediates non-transferrin-dependent delivery of iron	3.27	2.68	2.39
Rundc3b	RUN domain containing 3B; activated in breast cancer, correlates with metastasis	3.35	3.26	-2.32
Lix1l	Lix1-like; autophagy	2.01	1.69	-3.08
B2m	beta-2 microglobulin; Signaling by FGFR	0.5	0.5	-0.5
Hhip	Hedgehog-interacting protein	-3.01	-2.82	2.17
Prom2	prominin 2 membrane glycoproteins; localizes to basal epithelial cells may be involved in the organization of plasma membrane microdomains	-1.61	-1.53	1.26
Moxd1	monooxygenase, DBH-like 1; oxidoreductase activity	-4.34	-4.35	-4.42
Sfrp1	secreted frizzled-related protein 1; modulator of Wnt signaling	-2.08	-2.13	-2.19
Mir1983	microRNA 1983	-2.42	-2.52	-2.47
Pkia	protein kinase inhibitor, alpha	-2.15	-2.12	-2.39
Trp53i11	transformation related protein 53 inducible protein 11	-2.91	-2.39	-1.9
Serpinb5	serine or cysteine peptidase inhibitor, clade B, member 5; tumor suppressor	-4.11	-4.35	
Cxcr3	chemokine (C-X-C motif) receptor 3	-0.5		1.14
Ltf	lactotransferrin; iron homeostasis, regulation of cellular growth and differentiation and protection against cancer development and metastasis			3.82
Ankrd22	ankyrin repeat domain 22			3.43
Asb2	ankyrin repeat and SOCS box-containing 2; Antigen processing-Ubiquitination and Proteasome degradation			3.18
Emp3	epithelial membrane protein 3			-3.74
Sdpr	serum deprivation response			-2.78
Mapkapk3	mitogen-activated protein kinase- activated protein kinase 3			-1.49
Flt1	FMS-like tyrosine kinase 1; cell-surface receptor for VEGFA			-1.54

Supplementary Table S4. Expression of ABC transporters in PDT-resistant cell lines relative to 4T1 cells. TaqManArray qPCR data analyzed by GenEx software and normalized to Gapdh expression.

	KP1-1 vs 4T1)		KP1-2 vs 4T1		KP6-1 vs 4T1)		KP6-2 vs 4T1		T-1 vs 4T1		T-2 vs 4T1	
	Fold change	P-Value	Fold change	P-Value	Fold change	P-Value	Fold change	P-Value	Fold change	P-Value	Fold change	P-Value
**Abca1**	**183.440**	**0.004**	**7.009**	**0.001**	**139.989**	**0.000**	**151.867**	**0.001**	**n.d.**	**n.d.**	**1.010**	**0.927**
Abca2	1.033	0.915	1.944	0.086	1.190	0.739	1.379	0.043	1.271	0.042	1.698	0.016
Abca3	-1.414	0.111	1.025	0.850	-1.028	0.908	-1.322	0.124	-1.059	0.653	1.083	0.828
Abca7	1.584	0.104	-1.504	0.053	1.111	0.644	-1.785	0.104	-3.029	0.044	-1.573	0.112
Abcb10	-1.810	0.030	-2.062	0.008	-1.590	0.004	-2.175	0.107	1.861	0.009	2.534	0.005
Abcb11	n.d.	n.d.	-1.485	0.267	-2.053	0.114	n.d.	n.d.	n.d.	n.d.	-1.343	0.692
**Abcb1a**	**225.385**	**0.008**	**272.715**	**0.008**	**383.013**	**0.007**	**379.051**	**0.006**	**153.410**	**0.071**	**110.182**	**0.036**
Abcb6	1.526	0.328	1.882	0.302	1.552	0.335	1.102	0.795	-1.282	0.605	1.115	0.853
Abcb7	2.213	0.077	1.623	0.463	3.206	0.023	2.029	0.165	1.835	0.082	2.673	0.031
Abcb8	-1.740	0.003	1.040	0.892	-1.176	0.652	-1.770	0.065	-1.271	0.276	1.377	0.126
Abcc1	1.761	0.012	1.536	0.018	1.907	0.091	1.776	0.029	1.234	0.025	1.634	0.088
Abcc10	1.180	0.426	2.275	0.044	1.581	0.364	1.600	0.192	-1.156	0.455	1.625	0.091
Abcc3	1.111	0.846	1.148	0.800	2.890	0.118	3.013	0.180	-2.556	0.138	1.101	0.905
Abcc4	5.962	0.023	6.269	0.002	6.581	0.001	3.012	0.023	1.132	0.183	1.838	0.202
Abcc5	3.396	0.003	2.885	0.004	4.283	0.012	3.218	0.004	1.056	0.455	1.494	0.063
Abcc6	-1.205	0.791	2.284	0.599	-1.832	0.481	1.011	0.975	-1.196	0.613	-2.460	0.409
Abcc8	3.531	0.133	1.133	0.488	5.503	0.131	94.699	0.425	-3.248	0.034	n.d.	n.d.
Abcd1	2.602	0.026	2.173	0.036	3.094	0.041	2.345	0.005	1.090	0.436	1.747	0.295
Abcd3	1.690	0.463	1.878	0.026	1.504	0.056	1.265	0.149	-2.485	0.013	-1.025	0.833
Abcd4	3.076	0.019	1.504	0.119	3.024	0.038	2.773	0.073	1.604	0.107	1.944	0.225
Abce1	-1.783	0.263	-1.827	0.052	-1.661	0.058	-2.788	0.129	-1.099	0.134	1.724	0.007
Abcf1	1.108	0.667	1.210	0.582	1.364	0.119	-1.046	0.892	-1.301	0.110	1.275	0.126
Abcf2	-1.048	0.887	1.123	0.745	1.046	0.812	-1.347	0.654	1.055	0.802	1.733	0.164
**Abcg1**	**25.531**	**0.021**	**14.512**	**0.010**	**21.806**	**0.014**	**23.412**	**0.007**	**n.d.**	**n.d.**	**n.d.**	**n.d.**
Abcg2	-6.608	0.032	n.d.	n.d.	-37.185	0.010	n.d.	n.d.	2.029	0.252	5.146	0.031
Abcg4	-2.164	0.281	-1.140	0.857	-3.092	0.300	-5.422	0.066	-6.185	0.045	-1.746	0.325
Tap1	3.279	0.010	-1.138	0.802	4.297	0.052	3.502	0.013	-4.804	0.013	-2.284	0.252
Tap2	1.741	0.170	-1.219	0.614	1.363	0.053	-1.608	0.112	-4.371	0.076	-1.570	0.059

Supplementary Table S5. PDT response induced by different photosensitizers in parental MCF-7 and paclitaxel-resistant MCF-7/PacR cells. PDT response is expressed as LD_{50} (light dose killing 50 % cells at the given PS concentration) and the resistance index is defined as LD_{50} of resistant clone/LD_{50} of parental cells. Each value is a mean of at least three experiments.

Cell line	Challenge (PDT)	PDT response LD_{50} (J cm^{-2})	Resistance index
MCF-7	KP1 (0.7 μM)	1.11±0.08	1
	KP6 (1.6 μM)	0.95±0.16	1
	Temoporfin (1.0 μM)	7.25±1.63	1
MCF-7/PacR	KP1 (0.7 μM)	>13	>12
	KP6 (1.6 μM)	3.88±0.21	4
	Temoporfin (1.0 μM)	13.27±1.75	1.8
